# Prenatal maternal depression and child behavioural and developmental outcomes: an individual participant data meta-analysis in 76,514 children from the EU Child Cohort Network

**DOI:** 10.1016/j.lanepe.2026.101595

**Published:** 2026-01-29

**Authors:** Adriana P.C. Hermans, Demetris Avraam, Isabel K. Schuurmans, Ana G. Soares, Marius Lahti-Pulkkinen, Polina Girchenko, Tanja G.M. Vrijkotte, Susanne R. de Rooij, Ahmed Elhakeem, Judith van der Waerden, Barbara Heude, Chloé Vainqueur, Tiffany C. Yang, Rachael W. Cheung, Dan Lewer, Katrine Strandberg-Larsen, Tim Cadman, Maja Popovic, Francesca Candelora, Jari Lahti, Katri Räikkönen, Charlotte A.M. Cecil, Hanan El Marroun

**Affiliations:** a Generation R Study Group, Erasmus MC, University Medical Center Rotterdam, Rotterdam, the Netherlands; b Department of Child and Adolescent Psychiatry/Psychology, Erasmus MC University Medical Center – Sophia Children's Hospital, Rotterdam, the Netherlands; c Department of Public Health, Section of Epidemiology, University of Copenhagen, Copenhagen, Denmark; d MRC Integrative Epidemiology Unit at the University of Bristol, Bristol, UK; e Population Health Science, Bristol Medical School, University of Bristol, Bristol, UK; f University of Helsinki, Finland; g University of Edinburgh, United Kingdom; h Department of Psychology, Faculty of Medicine, University of Helsinki, Helsinki, Finland; i Clinical Medicine Research Unit, MRC Oulu, University of Oulu and Oulu University Hospital, Oulu, Finland; j Department of Public and Occupational Health, Amsterdam UMC, University of Amsterdam, Amsterdam, the Netherlands; k Amsterdam Public Health Research Institute, Amsterdam, the Netherlands; l Amsterdam Reproduction and Development Research Institute, Amsterdam, the Netherlands; m Department of Epidemiology and Data Science, Amsterdam UMC, University of Amsterdam, Amsterdam, the Netherlands; n Social Epidemiology, Mental health and Addictions team, Institut Pierre Louis d'Epidemiologie et de Santé Publique, INSERM, Sorbonne University, Paris, France; o Université Paris Cité and Université Sorbonne Paris Nord, Inserm, INRAE, Centre de Recherche en Épidémiologie et StatistiqueS (CRESS), Paris, France; p Bradford Centre for Health Data Science, Bradford Institute for Health Research, Bradford Teaching Hospitals NHS Foundation Trust, Bradford, United Kingdom; q Public Health Improvement, UK; r Cancer Epidemiology Unit, Department of Medical Sciences, University of Turin, CPO Piemonte, Turin, Italy; s Folkhälsan Research Centre, Folkhälsan, Helsinki, Finland; t Department of Obstetrics and Gynecology, Helsinki University Hospital, Helsinki, Finland; u Department of Psychology, Education and Child Studies, Erasmus School of Social and Behavioral Science, Erasmus University Rotterdam, Rotterdam, the Netherlands

**Keywords:** Prenatal depression, Behaviour, Mental health, Development, Meta-analysis

## Abstract

**Background:**

Prenatal maternal depression affects an estimated one in five women, with implications not only for the mother but also for the child, associating negatively with offspring mental health and cognition. This study aimed to investigate multiple outcomes within the same set of participants from multiple cohorts, explore sex-specific differences in associations, and examine of the role of timing of maternal depression.

**Methods:**

We performed large-scale individual participant data analyses with a sample size of up to 76,514 participants to investigate prospective associations between prenatal maternal depression and eight offspring behavioural and developmental outcomes, leveraging harmonised data from seven European birth cohorts. Cohort-specific estimates were combined using random-effects meta-analysis. Potential sex differences and the role of pre-pregnancy and postnatal depression in the associations were examined.

**Findings:**

Prenatal maternal depression was associated with higher internalising, externalising, attention deficit hyperactivity disorder, and autism spectrum disorder symptoms (6.61–10.90 increased percentile scores). Associations were similar between males and females, largely independent of pre-pregnancy depression, and partially mediated by postnatal maternal depression. Continuous prenatal depressive symptoms were associated with all eight offspring outcomes.

**Interpretation:**

These findings emphasise the importance of prenatal maternal depression as a key developmental risk factor. Future work should consider how best to support mental health during pregnancy and children exposed to prenatal depression. Our results contribute to the growing evidence underscoring the need for early intervention and tailored support for those experiencing depression during pregnancy.

**Funding:**

HappyMums Project, funded by the 10.13039/501100000780European Union (Grant Agreement n.101057390).

**Research in context**

**Evidence before this study**

We conducted a literature search for articles on prenatal maternal depression and offspring development in PubMed and Google Scholar and repeated searches in the period from January 2023 until August 2025. Search terms included “prenatal depression”, “maternal depression”, “antenatal depression”, “child development”, “behaviour”, “cognition”, “internalising problems”, “externalising problems”, “attention-deficit hyperactivity disorder” (ADHD), “autism spectrum disorder” (ASD), “motor”, “language”, and “non-verbal intelligence”. Prior research has shown that prenatal maternal depression is negatively associated with a range of developmental outcomes in children, across emotional, behavioural, and cognitive domains. However, previous meta-analyses were not performed using individual-level data and did not investigate the role of timing of maternal depression. In addition, prior studies have reported inconsistent findings on sex differences in the associations. Large-scale, harmonised investigations assessing multiple developmental domains within the same population are lacking.

**Added value of this study**

This study is the largest to date to assess prospective associations between prenatal maternal depression and multiple child behavioural and cognitive outcomes, using harmonised individual participant data from seven European cohorts, with a sample size of over 76,000 children. We examined eight developmental outcomes across a range of domains, tested for sex differences, and examined the role of pre-pregnancy and postnatal maternal depression. Our findings demonstrate associations between a binary measure of prenatal maternal depression and increased internalising, externalising, ADHD, and ASD symptoms as well as associations between prenatal depressive symptoms and all eight developmental outcomes.

**Implications of all the available evidence**

Our findings emphasise the role of prenatal maternal depression as a critical risk factor for differences in a broad spectrum of child developmental outcomes. The associations were comparable for male and female offspring, were largely independent of pre-pregnancy depression, and were partially mediated by postnatal depression. These results support the need for early identification and intervention strategies targeting maternal depression during pregnancy. Addressing maternal depression prenatally may offer a promising opportunity to promote healthy development in children.

## Introduction

Depression during pregnancy is a prevalent condition estimated to affect one in five pregnant women, with even higher prevalence in low- or lower-middle-income countries.^1^ Prenatal depression presents a burden and has potential risks for the expecting women.^2^ Moreover, it may have long-term consequences for the child regarding their cognitive, behavioural, and emotional development.^3^^,^^4^ A large meta-analysis showed consistent evidence for increased internalising and externalising problems during the first 18 years of life in offspring exposed to prenatal maternal depression.^4^ Systematic reviews convincingly show more mental health symptoms in children exposed to prenatal depression, reporting a five-fold higher risk of autism spectrum disorder (ASD) and an increased risk of attention deficit hyperactivity disorder (ADHD) symptoms.^5^^,^^6^ Studies have also reported changes in other developmental domains associated with exposure to prenatal maternal depression, such as decreased language and motor skills.^4^ Despite this body of research, the heterogeneity between studies is often too large to perform individual participant data meta-analysis.^6^ Moreover, the available meta-analyses have been limited to pooling estimates from different published studies without the possibility to uniformly control for potential confounding factors or explore potential mediating pathways.^3^, ^4^, ^5^ Furthermore, few studies focus on a range of outcomes across multiple domains of functioning, such as cognition, behaviour and mental health. This limits our ability to compare findings across outcomes and our understanding of symptom profiles of children prenatally exposed to maternal depression.

Foetal development is sexually dimorphic, with male and female foetuses showing different susceptibility to hormonal and inflammatory changes in the intrauterine environment.^7^ Given that hormonal and inflammatory changes may be mechanisms that explain the link between prenatal depression and child developmental problems, this link may show sex-specific susceptibility.^8^ Previous studies have presented considerable, yet often inconsistent, evidence for sex differences in the prospective associations between prenatal maternal depression and child psychological development. Quarini and colleagues found an increased risk of depression in adolescent girls compared to boys following prenatal exposure to maternal depressive symptoms.^9^ A meta-analysis by Ahun and colleagues found that maternal depression was associated with poorer cognitive development only in boys.^10^ For socioemotional problems, Maselko and colleagues reported a stronger association between prenatal maternal depression and total difficulties scores on the Strengths and Difficulties Questionnaire in boys.^11^ In contrast, some studies report no sex differences in the associations between prenatal depression and child outcomes.^12^ Understanding sex-specific symptom profiles could inform the development of more individualised risk detection strategies for children exposed to prenatal maternal depression.

Depression is often characterised by temporal stability and chronicity, with depressive symptoms persisting from the prenatal to the postnatal period.^13^ Investigating the role of timing is important; in the first place, maternal depression may relate to changes in offspring development through different pathways, such as changes in the in-utero environment during the prenatal period versus mechanisms related to mother-child bonding in the postnatal period.^8^^,^^14^ Secondly, because depression often reoccurs and can persist over time, it can be challenging to pinpoint what time point of exposure is most influential in these associations.^15^ For instance, research has shown that the effects of prenatal depression may to some extent be mediated by postnatal depression.^16^ However, many studies have investigated prenatal and postnatal depression separately, or have controlled for postnatal depression when investigating the role of prenatal depression.^4^^,^^6^^,^^17^ Furthermore, women with a history of depression are at increased risk of experiencing prenatal depression.^18^ Identifying the effects of pre-pregnancy, prenatal and postnatal depressive symptoms and formally investigating postnatal depression as a mediator in the associations between prenatal depression and child outcomes is informative for intervention studies and policy making. Thirdly, cumulative exposure to maternal depression at multiple time points could exacerbate its impact on offspring outcomes. Some studies that have investigated the effects of the chronicity of depression using repeated assessments of depression during pregnancy and after childbirth have shown that more chronic or cumulative exposure to maternal depression is associated with increased emotional problems in offspring, but the pre-pregnancy period was not included in these analyses.^13^^,^^19^, ^20^, ^21^

To address these gaps, we performed individual participant data meta-analyses using harmonised data from seven European birth cohorts to investigate: (I) the prospective associations between prenatal maternal depression and children's developmental outcomes from early childhood to adolescence, (II) potential sex differences in these associations, and (III) the role of the pre-pregnancy, prenatal, and postnatal period in the associations between maternal depression and offspring outcomes, and whether an increase in the number of time points of exposure to maternal depression would be associated with differences in developmental outcomes.

## Methods

This study's aims and analysis plans were preregistered in the Open Science Framework (OSF), for details, please see https://osf.io/kqcfy. Analysis plans were approved and published in August 2024 and first access to datasets was obtained January 2024.

### Study population

This study includes cohorts from the EU Child Cohort Network (ECCN), which brings together nineteen pregnancy and childhood cohorts with harmonised and standardised data.^22^ The ECCN provides a valuable resource that allows researchers to run identical analyses across multiple cohorts, ensuring consistent control for confounders and enabling comparable results. To be eligible for inclusion in this study, a cohort needed to have data on prenatal maternal depression and at least one of the eight outcome variables examined (see below). The following seven cohorts participated in this study, namely Amsterdam Born Children and their Development (ABCD), the Avon Longitudinal Study of Parents and Children (ALSPAC, ethics and population detailed in Supplementary Material), the Danish National Birth Cohort (DNBC), Étude des Déterminants pré et postnatals du développement et de la santé de l'Enfant (EDEN), the Generation R Study, Nascita e INFanzia: gli Effetti dell’Ambiente (NINFEA), and the Prediction and prevention of preeclampsia and intrauterine growth restriction study (PREDO) (not part of the ECCN). For recruitment details, please see Jaddoe and colleagues.^22^ The total sample size of this study included 76,514 children with complete data on exposure, covariates, and at least one outcome measure.

### Prenatal maternal depression

Prenatal maternal depression was assessed either by self-reported diagnosis (NINFEA, DNBC) or clinical questionnaires (ABCD, ALSPAC, EDEN, Generation R, PREDO). Based on these different measures, we developed a harmonisation protocol to extract a binary variable for depression in all cohorts. If prenatal depression was measured continuously (in ALSPAC, EDEN, Generation R, ABCD and PREDO), a validated cut-off was applied to create a binary measure of prenatal maternal depression, for details see Supplementary Table S1. Where available, the continuous measure was standardised (i.e., a mean of 0 and standard deviation of 1) to be used for sensitivity analyses to explore associations between the full range of depressive symptoms and child outcomes. Details on assessment tools and harmonisation steps can be found in the Supplementary.

### Offspring developmental outcomes

**Fig. 1:**
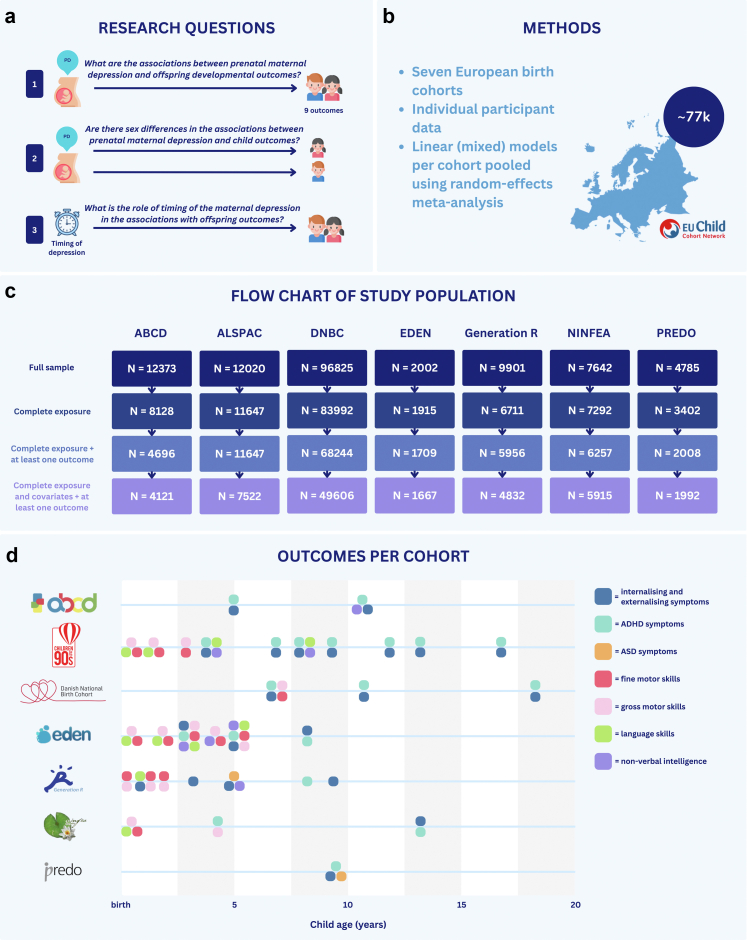
Overview of the study. *Note*: Panel a shows the three research questions, panel b shows the study's methods, panel c shows a flow chart of the included sample, and panel d shows the outcome data available per cohort (from top to bottom: ABCD, ALSPAC, DNBC, EDEN, Generation R, NINFEA, PREDO).

This study included eight outcome variables. Four outcomes related to mental health included internalising and externalising symptoms, ADHD symptoms, and ASD symptoms. Four motor and cognitive outcomes were fine and gross motor development, non-verbal intelligence, and language. All were assessed in offspring in childhood and adolescence (0–19 years). Harmonisation details have been reported elsewhere and are described in the online ECCN variable catalogue (https://data-catalogue.molgeniscloud.org/catalogue/catalogue/all/variables).^23^ Continuous percentile scores calculated within cohorts and age groups were used to allow comparisons across different scales. For example, internalising and externalising symptoms were assessed with the Strengths and Difficulties Questionnaire and Child Behaviour Checklist. These continuous scores per scale were converted into cohort-specific percentile scores (range: 0–100), calculated based on the distribution within each cohort. This transformation allowed comparability of symptom measures across instruments and cohorts. See Fig. 1d for details on the outcomes and assessment ages per cohort, information on the assessment tools can be found in the Supplementary. If an outcome was assessed repeatedly, all available measurements per child were included and analysed using mixed-effects models with a random intercept to account for within-child repeated measures (see Main analyses). All outcomes were based on parental report, except for non-verbal intelligence which was assessed by an examiner or a test performed by the child (see Supplemental Table S2 for details). As a deviation from the preregistration, we did not include EDEN in the ASD analyses, since this was assessed using the Alarm Distress Baby Scale at a very different age (0–1 years) than the other two cohorts Generation R (6 years) and PREDO (9 years). We also did not include PREDO in any motor, language, or non-verbal intelligence analyses, because their measures were not harmonisable with other cohorts.

### Covariates and mediators

Covariates included maternal age at birth (years), maternal smoking during pregnancy (yes/no), maternal alcohol consumption during pregnancy (yes/no), pre-pregnancy maternal body mass index (BMI, kg/m^2^), maternal country of birth (born in country of cohort/born abroad) (available in ABCD, ALSPAC, EDEN, Generation R, and NINFEA), maternal education (low/medium/high), child's assigned sex at birth (male/female) and age at outcome assessment (years). Pre-pregnancy depression (yes/no) (available in ALSPAC, DNBC, Generation R, NINFEA) was included as a confounder in one of the models to assess the associations of prenatal depression with offspring outcomes independent of pre-pregnancy depression. Different levels of covariate adjustment were explored and are described in Sensitivity analyses. Postnatal maternal depression within the first year after childbirth (yes/no) was analysed as a mediator (see Main analyses), again binarized at a clinical cut-off for cohorts with a continuous measure of the depression. Harmonisation details of all variables have been recorded in the online ECCN variable catalogue (https://data-catalogue.molgeniscloud.org/catalogue/catalogue/all/variables).

### Cumulative exposure to maternal depression

To assess the potential cumulative effect of exposure to maternal depression, we constructed a count variable that adds the binary measures of pre-pregnancy depression, prenatal depression, and postnatal depression. This variable was constructed only in cohorts with data on maternal depression harmonised at all three time points (ALSPAC, DNBC, Generation R, and NINFEA) and allowed up to one missing time point. This resulted in a cumulative score of maternal depression, weighted by the number of time points available, ranging from 0 to 3 irrespective of the timing of the exposure, with 0 indicating no depression during any time point and 3 indicating depression during all measured time points. This score was analysed as a continuous exposure variable.

### Statistical analyses

For all cohorts, children were included if they had complete data on the exposure (prenatal maternal depression), covariates, and at least one of the eight outcome measures. For sample descriptives, we presented proportions of categorical variables and means and standard deviations for continuous variables of both the included sample and the excluded sample. A deviation from our preregistration is that we performed complete-case analyses instead of imputing missing data, which was not feasible due to platform constraints. See Fig. 1 for an overview of the study methods. The GitHub repository can be found at: https://github.com/JanaHermans/prenatal-depression-meta-analysis. This study followed the Strengthening the Reporting of Observational Studies in Epidemiology (STROBE) reporting guideline.

#### Main analyses

Two-stage individual participant data meta-analysis was used to investigate prospective associations between prenatal maternal depression and offspring behavioural and developmental outcomes. In each individual cohort, we ran linear mixed models with random intercepts in case of repeated outcome data and linear regressions for single time point data. Effect estimates were combined by applying random-effects meta-analysis with the restricted maximum likelihood estimator. Random-effects meta-analysis allows for and estimates between-study variability, i.e., the true associations are assumed to differ per cohort. We investigated associations between a binary measure of prenatal maternal depression and each offspring outcome individually, adjusting for the covariates defined above. To explore potential sex differences, we additionally included an interaction term between prenatal maternal depression and the child's assigned sex at birth. To assess whether associations were independent of pre-pregnancy depression, we tested associations between prenatal maternal depression and offspring outcomes while additionally controlling for pre-pregnancy maternal depression. To explore whether the association between prenatal depression and the outcomes assessed was explained by postnatal depression, we investigated postnatal maternal depression as a mediator in the association between prenatal depression and offspring outcomes using causal mediation analysis.^24^ We estimated the natural indirect effect via postnatal depression and its 95% confidence intervals using 1000 bootstrap repetitions and calculated the proportion mediated by postnatal depression. For the mediation analyses, one outcome assessment was selected per child from the age range with the largest amount of data: 5–10 years for internalising and externalising symptoms, 3–9 years for ADHD symptoms, and 5–9 years for ASD symptoms. Finally, we assessed whether a weighted count variable of cumulative exposure to maternal depression at multiple time points (see Cumulative exposure to maternal depression) would be associated with developmental outcomes using a weighted score of maternal depression at multiple time points as the exposure variable. In other words, this tested whether an increase in the number of time points of exposure to maternal depression was associated with changes in offspring outcomes.

Analyses were performed in a federated analysis set-up using DataSHIELD (which mitigates disclosure risk of individual-level data) and scripts were shared with two cohorts (ALSPAC and PREDO) who did not have (all) data in DataSHIELD.^25^ Functions from the DataSHIELD dsBaseClient package (version 6.3.1) were used to fit the linear (mixed) models. Meta-analyses were performed using the “metafor” package (version 4.4-0) in R.^26^ Mediation analyses were performed using the “mediation” package. Heterogeneity across cohorts was reported by the p-value of the Cochran's Q test and the I-squared and τ-squared statistics. We applied false discovery rate (FDR) correction using the Benjamini-Hochberg method.

#### Sensitivity analyses

To check the robustness of our findings, we performed multiple sensitivity analyses. Firstly, we investigated associations between prenatal maternal depression and child outcomes in a basic model only controlling for the child's assigned sex at birth and age at outcome assessment for comparability to other studies without an extensive set of covariates. Second, to control for maternal national origin in the associations, we ran analyses additionally adjusting for maternal country of birth in cohorts for which this data was available (ABCD, ALSPAC, EDEN, Generation R, NINFEA). Third, we assessed associations between a continuous symptom score of prenatal maternal depression and child developmental outcomes in the five cohorts with a continuous measure of prenatal depressive symptoms (ABCD, ALSPAC, EDEN, Generation R, PREDO) to model the full symptom severity range and explore potential information loss in a binary measure of exposure. Lastly, we performed leave-one-out analyses to assess the impact of individual cohorts on our pooled results and explored shared-rater bias in Generation R, one of the largest cohorts with outcome data from multiple informants.

### Ethics approval

Ethical approval was obtained for all studies (see Supplementary for details).

### Role of the funding source

Funding sources played no role in the study design, data collection, data analysis, interpretation, or writing of the report.

## Results

Table 1 shows the study population characteristics per cohort. Mean maternal age at childbirth ranged from 27.0–33.2 years, assigned male sex at birth ranged from 49.6–52.4% (N = 24621–873), maternal country of birth outside the country of cohort ranged from 3.4–27.7% (N = 57–1337), and mothers with a high level of education ranged from 16.4–65.0% (N = 1234–1294). There were large differences in the proportion of women with prenatal maternal depression, varying from 0.7–21.1% (N = 365–421). Generally, this proportion was highest in cohorts that assessed prenatal maternal depression continuously using a validated symptom questionnaire (8.9–21.1%, N = 428–421). The lowest proportion was recorded in cohorts that assessed prenatal maternal depression using a binary self-reported measure (0.7–1.9%, N = 365–112). Assessment timing of prenatal depression varied by cohort (see Supplementary Table S1). The proportion of women with pre-pregnancy depression ranged from 2.7–19.0% (N = 1349–904). Characteristics of the excluded sample are described in Supplementary Table S4.

**Table 1:** Study characteristics.

	ABCD (the Netherlands) 2003–2004	ALSPAC (UK) 1991–1992	DNBC^a^ (Denmark) 1995–2002	EDEN (France) 2003–2006	Generation R (the Netherlands) 2002–2006	NINFEA (Italy) 2005–2016	PREDO (Finland) 2006–2010
*n* (% of original sample)	4121 (33.2)	7522 (62.6)	49606 (51.2)	1667 (83.3)	4832 (48.8)	5915 (77.4)	1992 (41.6)
Child characteristics							
Assigned sex, male, *n* (%)	2054 (49.8)	3818 (50.9)	24621 (49.6)	873 (52.4)	2401 (49.7)	3013 (50.9)	1008 (50.6)
Maternal characteristics							
Maternal age at childbirth, years, mean (SD)	32.1 (4.7)	27.0 (5.8)	30.2 (4.2)	29.7 (4.8)	31.0 (4.9)	33.2 (4.2)	32.0 (4.5)
Mother born abroad, yes, *n* (%)	987 (24.0, missing: 0.3%)	299 (4.2, missing: 5.9%)	–	57 (3.4, missing: 0.6%)	1337 (27.7, missing: 0.1%)	226 (3.8, missing: 0.0%)	–
High maternal education level, *n* (%)	2457 (59.6)	1234 (16.4)	24663 (49.7)	931 (55.8)	2377 (49.2)	3744 (63.3)	1294 (65.0)
Prenatal maternal depression, *n* (%)	699 (17.0)	1382 (18.4)	365 (0.7)	218 (13.1)	428 (8.9)	112 (1.9)	421 (21.1)
Postnatal maternal depression, *n* (%)	523 (14.3, missing: 11.3%)	554 (7.8, missing: 5.6%)	6325 (15.3, missing: 16.7%)	121 (8.2, missing: 11.6%)	230 (6.9, missing: 31.5%)	94 (2.5, missing: 36.5%)	317 (16.3, missing: 2.4%)
Pre-pregnancy depression, *n* (%)	–	603 (8.0, missing: 0.0%)	1349 (2.7, missing: 0.0%)	–	904 (19.0, missing: 1.5%)	190 (3.2, missing: 0.0%)	–
Maternal depression at more than one time points, *n* (%)	–	539 (7.2, missing: 0.0%)	612 (1.2, missing: 0.0%)	–	279 (5.8, missing: 0.4%)	82 (1.4, missing: 0.0%)	–
Any alcohol use in pregnancy, yes, n (%)	1553 (37.7)	5948 (79.1)	29439 (59.3)	853 (51.2)	2773 (57.4)	1728 (29.2)	312 (15.7)
Any smoking in pregnancy, yes, n (%)	436 (10.6)	1762 (23.4)	12064 (24.3)	419 (25.1)	1197 (24.8)	471 (8.0)	113 (5.7)
Pre-pregnancy BMI, mean (SD)	23.0 (3.9)	22.9 (3.8)	23.6 (4.2)	23.2 (4.6)	23.5 (4.2)	22.4 (3.8)	24.1 (4.6)

*Note*: Characteristics of the participating study population are based on complete data on the exposure, covariates, and at least one outcome. Calendar years below cohort names indicate the recruitment period. High maternal education level was classified as tertiary education. Total sample size = 76,514 children with complete data on exposure (prenatal maternal depression), covariates, and at least one outcome.

a Danish language skills sufficient to participate in computer-assisted telephone interviews were a prerequisite to enrol in the DNBC.

### Prenatal maternal depression and offspring developmental outcomes

#### Mental health-related outcomes

**Fig. 2:**
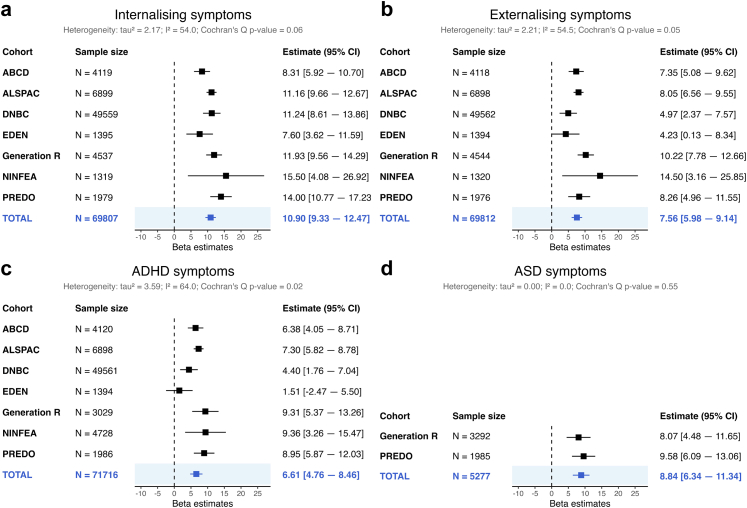
Forest plots showing the prospective associations between prenatal maternal depression and offspring mental health related outcomes. *Note*: Results shown are from two-stage individual participant data meta-analyses adjusted for maternal age at birth, maternal smoking during pregnancy, maternal alcohol consumption during pregnancy, pre-pregnancy maternal BMI, maternal education, the child's assigned sex at birth and age at outcome assessment. The column N refers to the number of observations included per analysis. Panel a shows results for internalising symptoms, panel b for externalising symptoms, panel c for ADHD symptoms, and panel d for ASD symptoms.

Prenatal maternal depression was associated with higher offspring internalising, externalising, ADHD and ASD symptoms. The meta-analyses including all seven cohorts showed that children exposed to prenatal depression on average had 10.90 (95% CI: 9.33, 12.47) higher percentile scores for internalising, 7.56 (95% CI: 5.98, 9.14) higher scores for externalising symptoms, and 6.61 (95% CI: 4.76, 8.46) higher ADHD scores, see Fig. 2. These analyses were adjusted for maternal age at birth, maternal smoking and alcohol consumption during pregnancy, pre-pregnancy maternal BMI, maternal education, the child's assigned sex at birth and age at outcome assessment. For ASD symptoms, available in Generation R and PREDO only, prenatal maternal depression was associated with 8.84 (95% CI: 6.34, 11.34) higher percentile scores. Heterogeneity statistics indicated low heterogeneity between cohorts for ASD symptoms (I^2^ = 0.0%) and moderate heterogeneity for internalising symptoms (I^2^ = 54.0%), externalising symptoms (I^2^ = 54.5%), and ADHD symptoms (I^2^ = 64.0%). Associations remained evident after correction for multiple testing (*P*_*adj*_ < *0.001* for all four outcomes).

#### Motor and cognitive outcomes

**Fig. 3:**
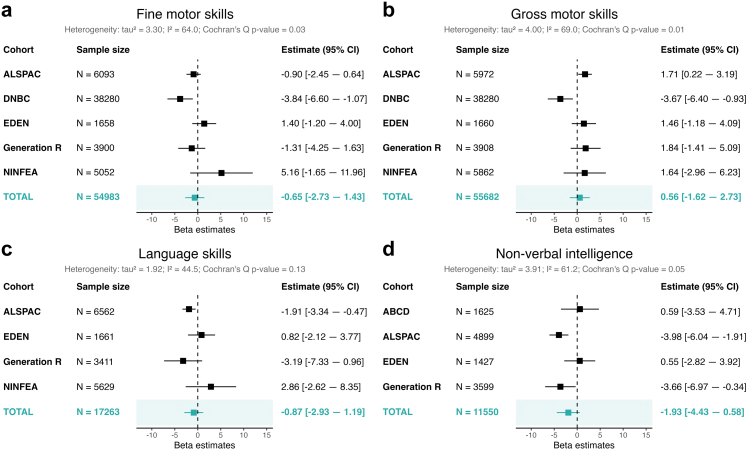
Forest plots showing the prospective associations between prenatal maternal depression and offspring motor and cognitive outcomes. *Note*: Results shown are from two-stage individual participant data meta-analyses adjusting for maternal age at birth, maternal smoking during pregnancy, maternal alcohol consumption during pregnancy, pre-pregnancy maternal BMI, maternal education, the child's assigned sex at birth and age at outcome assessment. The column N refers to the number of observations included per analysis. Panel a shows results for fine motor skills, panel b for gross motor skills, panel c for language skills, and panel d for non-verbal intelligence.

Little evidence was observed for associations with fine motor skills (adjusted mean difference in percentile scores between children exposed to prenatal depression = −0.65, 95% CI: [−2.73, 1.43]), gross motor skills (adjusted mean difference = 0.56, 95% CI: [−1.62, 2.73]), and language skills (adjusted mean difference = −0.87, 95% CI: [−2.93, 1.19]), see Fig. 3. For non-verbal intelligence, there was some suggestion of a negative association, but the confidence interval was wide and included the null (adjusted mean difference = −1.93, 95% CI: [−4.43, 0.58]). There was low heterogeneity for language skills (I^2^ = 44.5%) and moderate heterogeneity for the motor and non-verbal intelligence outcomes (I^2^ = 61.2%–69.0%).

### Sex differences

Overall, we found little evidence for interactions with the child's assigned sex for the association between prenatal maternal depression and offspring behaviour and developmental outcomes (see Supplementary Tables S5 and S6). Evidence of sex differences was only observed in one cohort (EDEN), namely less pronounced internalising percentile score increases in female sex assigned at birth, but these differences were no longer evident in the pooled estimates from all studies.

### Timing of maternal depression

**Fig. 4:**
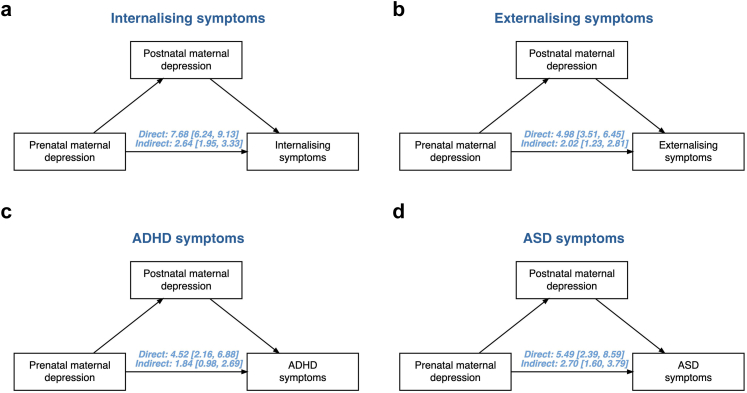
Mediation plots showing the associations between prenatal maternal depression and offspring outcomes through direct and indirect pathways via postnatal depression. *Note*: The results shown are pooled beta coefficients from random-effects meta-analyses and the 95% confidence intervals. The ASD estimates are from single-cohort analyses, namely in Generation R. Analyses were adjusted for maternal age at birth, maternal smoking and alcohol consumption during pregnancy, pre-pregnancy maternal BMI, maternal education, the child's assigned sex at birth and age at outcome assessment. The number of observations included was N = 46924 for internalising symptoms, N = 46910 for externalising symptoms, N = 46686 for ADHD symptoms, and N = 3804 for ASD symptoms. Cohort-specific estimates can be found in the Supplement. Panel a shows results for internalising symptoms, panel b for externalising symptoms, panel c for ADHD symptoms, and panel d for ASD symptoms.

When adjusting for maternal depression before pregnancy, prenatal maternal depression remained associated with higher internalising symptoms, externalising symptoms, ADHD symptoms, and ASD symptoms in children (see Table 2). When investigating postnatal maternal depression as a mediator, we found evidence for associations via both direct and indirect pathways of prenatal maternal depression with internalising, externalising, ADHD, and ASD symptoms, see Fig. 4. Proportion mediated was 25.6% for internalising symptoms, 28.1% for externalising symptoms, 28.2% for ADHD symptoms, and 32.4% for ASD symptoms, see Supplementary Tables S7–S10 for further details.

**Table 2:** Associations between prenatal maternal depression and offspring outcomes adjusting for pre-pregnancy depression.

	Internalising symptoms			Externalising symptoms			ADHD symptoms			ASD symptoms		
Cohort	N	β	95% CI	N	β	95% CI	N	β	95% CI	N	β	95% CI
ALSPAC	6899	10.38	[8.85, 11.90]	6898	7.47	[5.95, 8.99]	6898	6.75	[5.25, 8.26]			
DNBC	49555	6.61	[3.79, 9.42]	49558	3.26	[0.47, 6.05]	49557	2.48	[−0.35, 5.32]			
GenR	4474	10.04	[7.60, 12.47]	4481	8.75	[6.23, 11.27]	2995	7.64	[3.59, 11.68]	3256	7.28	[3.57, 10.99]
NINFEA	1318	12.58	[−1.11, 26.27]	1319	9.25	[−4.34, 22.85]	4727	4.11	[−2.90, 11.13]			
**TOTAL**	**62246**	**9.31**	**[7.23, 11.40]**	**62256**	**6.71**	**[3.82, 9.59]**	**64177**	**5.42**	**[2.82, 8.02]**	**3256**	**7.28**	**[3.57, 10.99]**

*Note*: Models were adjusted for maternal age at birth, maternal smoking and alcohol consumption during pregnancy, pre-pregnancy maternal BMI, maternal education, pre-pregnancy maternal depression, the child's assigned sex at birth and age at outcome assessment. The column N refers to the number of observations included per analysis. The I-squared statistic per analysis was: 54.0% for internalising symptoms, 73.9% for externalising symptoms, 61.1% for ADHD symptoms.

Furthermore, each additional time point of exposure to maternal depression was associated with a 4.43 (95% CI: [1.03, 7.83]) increase in internalising symptoms, a 3.22 (95% CI: [0.56, 5.87]) increase in externalising symptoms, and a 3.03 (95% CI: [1.69, 4.37]) increase in ASD symptoms (Generation R only), see Table 3. Although there was also an increase in ADHD symptoms, the confidence interval spanned the null (adjusted mean difference = 2.36, 95% CI: [−0.45, 5.17]).

**Table 3:** Associations between cumulative exposure to maternal depression and offspring outcomes.

	Internalising symptoms			Externalising symptoms			ADHD symptoms			ASD symptoms		
Cohort	N	β	95% CI	N	β	95% CI	N	β	95% CI	N	β	95% CI
ALSPAC	6899	7.80	[6.91, 8.68]	6898	5.57	[4.69, 6.46]	6898	4.98	[4.11, 5.85]			
DNBC	47098	0.20	[−0.32, 0.71]	47099	0.12	[−0.39, 0.63]	47098	0.23	[−0.29, 0.74]			
GenR	4568	5.79	[4.90, 6.68]	4574	4.70	[3.78, 5.63]	3052	4.60	[3.14, 6.07]	3314	3.03	[1.69, 4.37]
NINFEA	1265	3.84	[−0.14, 7.82]	1266	2.15	[−1.78, 6.09]	4572	−0.57	[−2.69, 1.56]			
**TOTAL**	**59830**	**4.43**	**[1.03, 7.83]**	**59837**	**3.22**	**[0.56, 5.87]**	**61620**	**2.36**	**[−0.45, 5.17]**	**3314**	**3.03**	**[1.69, 4.37]**

*Note*: Models were adjusted for maternal age at birth, maternal smoking and alcohol consumption during pregnancy, pre-pregnancy maternal BMI, maternal education, the child's assigned sex at birth and age at outcome assessment. *β* estimates indicate the increase in offspring outcome percentile scores with the addition of one time point of maternal depression. The column N refers to the number of observations included per analysis. The I-squared statistic per analysis was: 98.1% for internalising symptoms, 96.7% for externalising symptoms, 96.7% for ADHD symptoms.

### Sensitivity analyses

When additionally controlling for maternal country of birth in the five cohorts with data on this variable, the results remained similar to the main analysis (see Supplementary Tables S13 and S14). Sensitivity analyses with continuous prenatal depressive symptoms in the five cohorts with this measure showed associations in the same direction as the ones observed with the binary exposure, but now evidence of an association was observed with all child developmental outcomes (see Supplementary Tables S15 and S16). Leave-one-out analyses showed that the main findings for internalising, externalising, ADHD, and ASD symptoms were robust against the changes associated with iteratively leaving out single cohorts (see Supplementary Table S19). For the remaining outcomes, leave-one-out analyses showed an association for increased gross motor skills when leaving out DNBC (*β* = 1.67, 95% CI: [0.51, 2.84]), and decreased language skills (*β* = −1.76, 95% CI: [−3.08, −0.44]) and non-verbal intelligence (*β* = −2.82, 95% CI: [−5.25, −0.39]) when leaving out EDEN (see Supplementary Table S20). Lastly, prenatal maternal depression was associated with teacher-rated ADHD symptoms, although the strength of association was smaller than for maternal-reported outcomes (see Supplementary Table S21).

## Discussion

In this study, we performed individual-participant meta-analyses with data from 76,514 children from seven cohorts across Europe to examine developmental outcomes in offspring exposed to prenatal maternal depression. Analyses were adjusted for the same set of covariates across all cohorts, aiding interpretability and comparability. A broad range of harmonised outcomes was investigated, employing a comprehensive approach to characterising offspring symptom profiles associated with exposure to prenatal maternal depression. Further, we investigated whether associations vary by offspring sex and timing of maternal depression, taking into account the role of pre-pregnancy and postnatal depression. We highlight three key findings; firstly, a binary measure of maternal depression was robustly associated with increased mental health-related outcomes in offspring, but not motor and cognitive outcomes. Continuous prenatal depressive symptoms were associated with all eight offspring outcomes. Secondly, associations were not moderated by the child's assigned sex at birth. Lastly, the associations were independent of pre-pregnancy maternal depression and partly mediated by postnatal maternal depression. For some child outcomes, effect sizes cumulatively increased based on the chronicity of maternal depression.

This study showed that children exposed to prenatal maternal depression on average have six to ten percentile points more internalising, externalising, ADHD and ASD symptoms than unexposed children. This may indicate that exposed children experience a higher risk of developing mental health problems, especially since research has shown that half of individuals with a lifetime mental diagnosis had their onset before the age of 18 years.^27^ The findings align with previous research, such as a meta-analysis by Rogers and colleagues in a sample of 195,751 children aged 0–18 years which showed prospective associations between prenatal maternal depression and offspring internalising and externalising symptoms.^4^ A recent study by Lin and colleagues also reported associations between parental depression and offspring neurodevelopmental disorders, including ADHD and ASD.^28^ Findings from other studies are only partly in line with the current results; Szekely and colleagues reported that maternal general affective symptoms during pregnancy were associated with general psychopathology and with specific internalising but not with specific externalising problems.^29^ This discrepancy may be due to the slight difference in exposure, namely general affective symptoms in contrast to depression in the present study. In addition, the outcomes were assessed differently, since Szekely and colleagues performed factor analysis to extract a general factor of psychopathology and domain-specific factors of internalising and specific externalising symptoms, while we used overall internalising and externalising symptoms as our outcome domains.

Our study found that a binary measure of prenatal maternal depression was not consistently associated with motor, language, or non-verbal intelligence outcomes. Similar to this study, recent work by Cragoe and colleagues also reported no evidence for associations between prenatal maternal depression and offspring communication, gross motor, and fine motor outcomes.^30^ However, this is in contrast with the work of Rogers and colleagues who reported poorer language and motor development associated with prenatal exposure to maternal depression.^4^ A reason for this discrepancy could be the larger sample size of their meta-analyses as well as a methodological difference, namely that the current study conducted an individual-level participant data meta-analysis as opposed to pooling effect estimates from existing studies. Importantly, meta-analysis of effect estimates from published work does not allow alignment of analytic strategies, such as harmonisation of all included variables and adjustment for the same set of covariates in the models. Of note, sensitivity analyses in five cohorts with continuous exposure data showed that continuous prenatal depressive symptoms were associated with all eight offspring outcomes in this study. This may reflect the impact of greater heterogeneity of the binary exposure assessment, result from a different selection of included cohorts, or indicate that the associations with some offspring outcomes may be better captured using a continuous symptom scale. Altogether, this study showed some evidence for associations between prenatal depression and motor skills, language skills, and non-verbal intelligence, albeit not as consistently as the outcomes related to emotional and neurodevelopmental outcomes.

Previous studies have presented considerable, yet often inconsistent, evidence of sex-specific differences in the associations between prenatal maternal depression and offspring behavioural and emotional development.^7^ Some studies report stronger associations between prenatal maternal depression and child outcomes in girls, some show stronger associations in boys, and others report no sex differences in the associations.^9^^,^^11^^,^^12^ In our study, we showed little evidence of moderation of the associations by the child's assigned sex at birth when results were meta-analysed, with only one individual cohort showing stronger associations in males for internalising symptoms.

Limited research has been devoted to studying the effects of timing of maternal depression on the associations with offspring development. Here we address this and show that the associations of prenatal maternal depression with offspring internalising, externalising, ADHD, and ASD symptoms were independent of pre-pregnancy depression. This finding highlights that the associations may at least to some extent reflect the importance of the intrauterine environment, where the foundations for foetal brain development occur. Similar to previous research, this study found that prenatal maternal depression is associated with increased mental health-related symptoms in offspring both via direct pathways and indirect pathways mediated by postnatal depression.^16^ This shows that observed associations are not exclusively explained by the intrauterine environment, but may be partly related to the postnatal environment. Findings further show that more cumulative exposure to maternal depression across time periods was associated with increased risk of internalising, externalising, and ASD symptoms. This finding aligns with previous studies that reported chronic or increased exposure to maternal depression is linked to poorer socioemotional outcomes in offspring.^13^^,^^19^, ^20^, ^21^ Altogether, early intervention on maternal depression may have benefits not only for the mother but also for the child. Furthermore, the novel finding that most developmental changes associated with prenatal depression occur independently of pre-pregnancy depression highlights the importance of the prenatal period for screening of such symptoms.

Sensitivity analyses showed that the main findings of the prospective associations between prenatal maternal depression and internalising, externalising, ADHD, and ASD symptoms were robust to different levels of covariate adjustment, continuous versus binary measurement of exposure, and leave-one-out analyses. The sensitivity analyses showed that continuous measures of maternal depressive symptoms were associated with all outcomes, which could suggest that this dimensionality may more accurately capture meaningful variation relevant to offspring development in the general population. The lack of association in the primary analysis with motor skills, language, and non-verbal intelligence may stem from heterogeneity and reduced sensitivity of binary indicators across diverse cohorts. Some leave-one-out analyses and analyses adjusting for maternal country of birth showed additional associations with other outcomes. However, it is likely that these results are related to the smaller set of cohorts analysed (due to lack of data availability on all variables included in the sensitivity analyses). For instance, the association between prenatal depression and increased gross motor skills when leaving out DNBC may be related to the fact that this outcome was assessed at a later age in DNBC (mean age 7.0 years) compared to the other cohorts (mean age 1.5–2.8 years).

Several limitations of this study need to be mentioned. The required multi-cohort coordination did not allow for the mitigation of potential selection bias using methods like imputation or full information maximum likelihood. Furthermore, despite not all cohorts relying on maternal reports for both exposure and outcome data, potential effects of shared-rater bias could not formally be tested using the available harmonised data. However, sensitivity analyses in Generation R showed prenatal depression remained associated, albeit to a smaller extent, with teacher-reported ADHD outcomes. In addition, given the lack of harmonised genetic data in the ECCN, this study could not account for potential genetic confounding to test the role of shared genetic influences in the observed associations. While this study finds evidence for robust associations between prenatal maternal depression and offspring psychological development, underlying mechanisms are not explored. Future research applying genetically informed approaches is needed to better capture potential gene-environment interplay and the role of family environment in the observed associations, for instance using trio-designs and sibling cohorts. Another limitation is the heterogeneity of exposure assessments across cohorts, which we aimed to tackle with sensitivity analyses using leave-one-out analyses and restricting analyses to cohorts with a similar continuous measure of prenatal depression. Furthermore, in some cohorts, ADHD symptoms were assessed with instruments only using an attention scale, thereby missing representation of the hyperactivity and impulsivity dimensions. Furthermore, in some cohorts the attention problems scale was part of both the externalising and ADHD outcomes. Hence, this partial overlap should be considered when interpreting findings for these outcomes. Moreover, only two cohorts included in this study had data on ASD symptoms. Future research performing individual-level participant data meta-analysis in more than two cohorts is necessary to confirm the current findings of increased ASD symptoms. Given that the role of prenatal antidepressant use in the observed associations could not be studied, future meta-analyses in studies with harmonised data on prenatal antidepressant use are needed to appropriately study the independent associations of prenatal maternal depression with offspring outcomes. Lastly, the current study is restricted to meta-analyses of European cohorts. Findings need to be validated in cohorts outside of Europe, especially given the higher prevalence of prenatal maternal depression in low- or lower-middle-income countries.^1^ Future research should investigate trimester-specific associations between maternal depression and offspring development, since effects may vary depending on the stage of foetal development. Furthermore, future studies should explore potential lifestyle and environmental factors that may exacerbate or dampen the observed associations between prenatal maternal depression and offspring development. Such research could identify modifiable intervention targets to reduce risk in exposed offspring.

In conclusion, prenatal maternal depression may have long-lasting consequences for offspring psychological development. This study found robust associations between prenatal maternal depression and increased internalising, externalising, ADHD, and ASD symptoms in offspring. The prenatal period emerged as an important period of exposure, even when exploring the role of pre-pregnancy and postnatal maternal depression. With about one in five pregnant people estimated to experience depression during pregnancy, this study has important implications for the development of interventions to reduce prenatal maternal depression and for future research to study potential modifiable factors that may provide parents and children with support and promote resilience in offspring development.

## Contributors

Adriana Hermans was responsible for conceptualisation, formal analysis, investigation, visualisation, data curation, and writing–original draft. Demetris Avraam contributed to formal analysis and software, writing–review and editing. Isabel Schuurmans contributed to writing–review and editing. Ana Goncalves Soares contributed to data analysis in ALSPAC, interpretation of the results, and review and editing of the manuscript. Marius Lahti-Pulkkinen contributed to conceptualisation, data curation, formal analysis, funding acquisition, investigation, methodology and writing–review and editing. Polina Girchenko contributed to data curation and data analysis in the PREDO cohort, and to writing: review and editing of the original text. Tanja Vrijkotte contributed to data collection, data interpretation and writing. Susanne de Rooij contributed to writing–review and editing. Ahmed Elhakeem contributed to data curation, project administration, and writing–review. Chloé Vainqueur contributed to data curation: creating variables and making data available. Rachael Cheung contributed to methodology, writing–review and editing. Dan Lewer contributed to data curation and writing–review and editing. Katrine Strandberg-Larsen contributed to harmonisation efforts, the analysis plan, verifying the DNBC data and interpretation of results. Maja Popovic contributed to data collection and curation, data interpretation writing–review and editing. Francesca Candelora contributed to writing –review and editing. Jari Lahti participated in the designing and funding acquisition of the PREDO study, their data collection, and statistical analyses, as well as reviewing and editing the manuscript. Katri Räikkönen contributed to conceptualisation, data curation, formal analysis, funding acquisition, investigation, methodology, project administration, resources, supervision, writing–review and editing. Charlotte Cecil contributed to conceptualisation, funding acquisition, investigation, supervision, and writing–review and editing. Hanan El Marroun was involved in conceptualisation, data curation (harmonisation), funding acquisition, methodology, project administration, supervision, and writing–review and editing. Adriana Hermans, Demetris Avraam, Ana Goncalves Soares, Marius Lahti-Pulkkinen, Polina Girchenko, Chloé Vainqueur, and Maja Popovic have accessed and verified the underlying data. Adriana Hermans and Hanan El Marroun were responsible for the decision to submit the manuscript for publication.

## Data sharing statement

The data used in this study is not publicly available, as access is controlled by each individual cohort. To obtain the data, separate requests would need to be made to each respective cohort.

## Declaration of interests

The authors declare no conflict of interest.
