# Supplementary material for: Prenatal maternal depression and child behavioural and developmental outcomes: an individual participant data meta-analysis in 76,514 children from the EU Child Cohort Network
Source: Lancet Reg Health Eur. 2026 Jan 29;63:101595. doi: 10.1016/j.lanepe.2026.101595 (PMC12870471; doi:10.1016/j.lanepe.2026.101595)
Supplement: Supplementary Material [file mmc1.docx]

**Supplementary material**

Table of Contents

[Details on study population 2](#_Toc216869038)

[Cohort and author specific acknowledgements and funding 4](#_Toc216869039)

[Table S1. Assessment tools for exposure measures per cohort 6](#_Toc216869040)

[Table S2. Assessment tools for outcome measures per cohort 7](#_Toc216869041)

[Table S3. Study characteristics – outcome variables 8](#_Toc216869042)

[Table S4. Study characteristics of excluded participants per cohort due to missing data on exposure, outcomes and/or confounders. 10](#_Toc216869043)

[Table S5. Interaction between prenatal maternal depression and child’s assigned sex at birth – internalising, externalising, ADHD and ASD outcomes. 11](#_Toc216869044)

[Table S6. Interaction between prenatal maternal depression and child’s assigned sex at birth – motor, language, and non-verbal intelligence outcomes. 12](#_Toc216869045)

[Table S7. Associations between prenatal depression and internalising symptoms via direct and indirect pathways mediated by postnatal depression. 13](#_Toc216869046)

[Table S8. Associations between prenatal depression and externalising symptoms via direct and indirect pathways mediated by postnatal depression. 14](#_Toc216869047)

[Table S9. Associations between prenatal depression and ADHD symptoms via direct and indirect pathways mediated by postnatal depression. 15](#_Toc216869048)

[Table S10. Associations between prenatal depression and ASD symptoms via direct and indirect pathways mediated by postnatal depression. 16](#_Toc216869049)

[Table S11. Association between prenatal maternal depression and internalising, externalising, ADHD and ASD outcomes, adjusted for assigned sex and age at outcome assessment. 17](#_Toc216869050)

[Table S12. Associations between prenatal maternal depression and motor, language, and non-verbal intelligence outcomes, adjusted for assigned sex and age at outcome assessment. 18](#_Toc216869051)

[Table S13. Associations between prenatal maternal depression and internalising, externalising, ADHD and ASD outcomes, additionally adjusted for maternal country of birth. 19](#_Toc216869052)

[Table S14. Associations between prenatal maternal depression and motor, language, and non-verbal intelligence outcomes, additionally adjusted for maternal country of birth. 20](#_Toc216869053)

[Table S15. Associations between continuous prenatal maternal depressive symptoms and internalising, externalising, ADHD and ASD outcomes. 21](#_Toc216869054)

[Table S16. Associations between continuous prenatal maternal depressive symptoms and motor, language, and non-verbal intelligence outcomes. 22](#_Toc216869055)

[Table S17. Individual associations between pre-pregnancy depression and internalising, externalising, ADHD and ASD outcomes. 23](#_Toc216869056)

[Table S18. Individual associations between postnatal depression and internalising, externalising, ADHD and ASD outcomes. 24](#_Toc216869057)

[Table S19. Leave-one-out analyses for the associations between prenatal depression and internalising, externalising, ADHD, and ASD symptoms. 25](#_Toc216869058)

[Table S20. Leave-one-out analyses for the associations between prenatal depression and motor, language, and non-verbal intelligence outcomes. 26](#_Toc216869059)

[Table S21. Associations between prenatal maternal depression ADHD outcomes based on maternal and teacher report (Generation R only). 27](#_Toc216869060)

[References 28](#_Toc216869061)

# Details on study population

Data for this study are part of the EU Child Cohort Network (ECCN), which is a Horizon2020-funded LifeCycle Project that has brought together nineteen pregnancy and childhood cohorts with harmonised and standardised data. Details have been reported elsewhere (https://doi.org/10.1007/s10654-020-00662-z). The cohorts included in the analyses include Amsterdam Born Children and their Development (ABCD), Avon Longitudinal Study of Parents and Children (ALSPAC), Danish National Birth Cohort (DNBC), Étude des Déterminants pré et postnatals du développement et de la santé de l'Enfant (EDEN), the Generation R study, and Nascita e INFanzia: gli Effetti dell’Ambiente (NINFEA). One cohort outside the ECCN was included in the analyses, namely the Prediction and prevention of preeclampsia and intrauterine growth restriction study (PREDO). Participants in the ABCD cohort were recruited in the Netherlands between 2003 and 2004, details have been reported by van Eijsden and colleagues.^1^ Ethical approval was obtained from the Central Committee on Research Involving Human Subjects in The Netherlands, the medical ethics review committees of the participating hospitals and the Registration Committee of the Municipality of Amsterdam. Participants in the DNBC cohort were recruited in Denmark between 1995 and 2002, details have been reported by Strandberg-Larsen and colleagues.^2^ Ethical approval was obtained from the Danish National Committee on Health Research Ethics and Danish Data Protection Agency. The regional scientific ethical Committee for the municipalities of Copenhagen and Frederiksberg approved the study on the 8th of February 1995, project (KF) 01-471/94. Data handling in the DNBC was approved by Statens Serum Institut (SSI) under ref. no 18/04608 and is covered by the general approval (Fællesanmeldelse) given to SSI. The cohort was approved under ref. no 2008-54-0431. Written informed consent was obtained from all parents and children before enrolment. Participants in the EDEN cohort were recruited in France between 2003 and 2006, details have been reported by Heude and colleagues.^3^ Ethical approval was obtained on the 12th of December 2002 from the ethics committee (CCPPRB, N°02-70) of Kremlin Bicêtre and from the Commission Nationale Informatique et Liberté (CNIL, n°902267), the French data privacy institution. Written informed consent was obtained twice from parents, once at enrolment and once after the child’s birth. Participants in the Generation R Study were recruited in the Netherlands between 2002 and 2006, details have been reported by Kooijman and colleagues.^4^ Ethical approval was obtained from The Medical Ethics Committee of Erasmus Medical Center approved the study (MEC 198.782/2001/31, MEC 217.595/2002/202, MEC-2007-413; NL21545.078.08, MEC-2012-165; NL40020.078.12 en MEC 2015-749; NL55105.078.15). Written informed consent was provided by all parents and children. Participants in the NINFEA cohort were recruited in Italy between 2005 and 2016, details have been reported by Richiardi and colleagues.^5^ Ethical approval was obtained from Ethical Committee of the San Giovanni Battista Hospital and CTO/CRF/Maria Adelaide Hospital of Turin (approval N.0048362 and following amendments) in 2005 and written informed consent from participants was obtained at enrolment. Participants in the PREDO cohort were recruited in Finland between 2006 and 2010, details have been reported by Girchenko and colleagues.^6^ Ethical approval was obtained from Ethics Committee of Obstetrics and Gynaecology and Women, Children and Psychiatry of the Helsinki and Uusimaa Hospital District and by the participating hospitals. All participants provided written informed consent, and consent of participating children was provided by parent(s)/guardian(s). The study has been registered as ClinicalTrials.gov identifier ISRCTN14030412. For the ALSPAC cohort, pregnant women resident in Avon, UK with expected dates of delivery between 1st April 1991 and 31st December 1992 were invited to take part in the study. 20,248 pregnancies have been identified as being eligible and the initial number of pregnancies enrolled was 14,541. Of the initial pregnancies, there was a total of 14,676 foetuses, resulting in 14,062 live births and 13,988 children who were alive at 1 year of age. The total sample size for analyses using any data collected after the age of seven is therefore 15,454 pregnancies, resulting in 15,658 foetuses. Of these 14,901 children were alive at 1 year of age. See the work by Boyd and colleagues and Fraser and colleagues for details.^7,8^ Please note that the study website contains details of all the data that is available through a fully searchable data dictionary and variable search tool, see <http://www.bristol.ac.uk/alspac/researchers/our-data/>. Ethical approval for the study was obtained from the ALSPAC Ethics and Law Committee and the Local Research Ethics Committees on the 28^th^ of November 1989. Informed consent for the use of all data collected was obtained from participants following the recommendations of the ALSPAC Ethics and Law Committee at the time. Participants can contact the study team at any time to retrospectively withdraw consent for their data to be used. Study participation is voluntary and during all data collection sweeps, information was provided on the intended use of data. The completion of a questionnaire, either on paper or online, was considered to be written consent from participants to use their data for research purposes. For further details, see <https://www.bristol.ac.uk/alspac/researchers/research-ethics/>. The informed consent obtained from ALSPAC (Avon Longitudinal Study of Parents and Children) participants does not allow the data to be made available through any third party maintained public repository. Supporting data are available from ALSPAC on request under the approved proposal number, B4580. Full instructions for applying for data access can be found here: <http://www.bristol.ac.uk/alspac/researchers/access/>. The ALSPAC study website contains details of all available data (<http://www.bristol.ac.uk/alspac/researchers/our-data/>).

# Cohort and author specific acknowledgements and funding

The general design of the Generation R Study is made possible by financial support from the Erasmus MC, University Medical Center, Rotterdam, Erasmus University Rotterdam, Netherlands Organization for Health Research and Development (ZonMw), Netherlands Organisation for Scientific Research (NWO), Ministry of Health, Welfare and Sport and Ministry of Youth and Families. This project received funding from the European Union's Horizon 2020 research and innovation programme (LIFECYCLE, grant agreement No 733206, 2016; EUCAN-Connect grant agreement No 824989; ATHLETE, grant agreement No 874583, LongITools grant No 874739). VJ received funding from a Consolidator Grant from the European Research Council (ERC-2014-CoG-648916). The authors gratefully acknowledge the contribution of participants, research collaborators, general practitioners, hospitals, midwives, and pharmacies in Rotterdam. The work of Dr. Charlotte Cecil is supported the European Union’s HorizonEurope Research and Innovation Programme (FAMILY, grant agreement No 101057529; HappyMums, grant agreement No 101057390) and the European Research Council (TEMPO; grant agreement No 101039672). This research was conducted while CAMC was a Hevolution/AFAR New Investigator Awardee in Aging Biology and Geroscience Research. The work by professor Hanan El Marroun was supported by Stichting Volksbond Rotterdam, grant 015.016.056 from the Netherlands Organisation for Scientific Research (NWO) Aspasia, grant 101057390 from the European Union’s Horizon Research and Innovation Program (HappyMums), and by grant 953327 from the Marie Skłodowska-Curie Actions Innovative Training Networks program (Serotonin and Beyond). Born in Bradford receives funding from by a joint grant from the UK Medical Research Council (MRC) and UK Economic and Social Science Research Council (ESRC) [MR/N024391/1]; the British Heart Foundation [CS/16/4/32482]; a Wellcome Infrastructure Grant [WT101597MA]; The National Institute for Health Research under its Applied Research Collaboration for Yorkshire and Humber [NIHR200166]; Wellcome Trust Discovery Award [310017/Z/24/Z]; UKRI Population Health Improvement UK Healthy Urban Places consortium (MR/Y022785/1). The views expressed are those of the author(s), and not necessarily those of the NHS, the NIHR or the Department of Health and Social Care. Dr. Girchenko and Dr. Lahti-Pulkkinen received financial support from the Research Council of Finland (Research Fellow project No. 354990 and No. 330206 respectively). Professor Lahti received financial support from the Strategic Research Council (SRC) established within the Academy of Finland (decision numbers: 352700 and 372317), The Finnish Society for Sciences and Letters, and the Finnish Cultural Foundation. Professor Katri Räikkönen received funding from the European Union’s Horizon Europe research and innovation programme [Grant Agreement n.101057390; HappyMums] and HiLife Fellows Programme 2023-2025. Dr. Ana Soares works in a unit supported by the University of Bristol and the UK Medical Research Council (MC_UU_00032/5) and was supported by the European Union’s Horizon 2020 research and innovation programme (grant agreement No 874739, LongITools). The UK Medical Research Council and Wellcome (Grant ref: MR/Z505924/1) and the University of Bristol provide core support for ALSPAC. This publication is the work of the authors and they will serve as guarantors for the contents of this paper. We are extremely grateful to all the families who took part in this study, the midwives for their help in recruiting them, and the whole ALSPAC team, which includes data collection staff, data and administrations staff, technical managers and the technical staff with the Bristol Bioresrource Laboratory, based within the University of Bristol. The EDEN study was supported by Foundation for medical research (FRM), National Agency for Research (ANR), National Institute for Research in Public health (IRESP: TGIR cohorte santé 2008 program), French Ministry of Health (DGS), French Ministry of Research, INSERM Bone and Joint Diseases National Research (PRO-A), and Human Nutrition National Research Programs, Paris-Sud University, Nestlé, French National Institute for Population Health Surveillance (InVS), French National Institute for Health Education (INPES), the European Union FP7 programmes (FP7/2007–2013, HELIX, ESCAPE, ENRIECO, Medall projects), Diabetes National Research Program (through a collaboration with the French Association of Diabetic Patients (AFD)), French Agency for Environmental Health Safety (now ANSES), Mutuelle Générale de l’Education Nationale a complementary health insurance (MGEN), French national agency for food security, French-speaking association for the study of diabetes and metabolism (ALFEDIAM). The ABCD study was financially supported by the Netherlands Organization for Health Research and Development (grants 21000076, 92003489, and 040-00812-98-11010), Dutch Heart Foundation (grant 2007B103) and Sarphati Amsterdam. The authors thank all participating hospitals, obstetric clinics, and general practitioners for their assistance in implementing the ABCD study, and also thank the ABCD-youth and their parents for their cooperation.

| **Cohort** | **Prenatal maternal depression** | **Pre-pregnancy maternal depression** | **Postnatal maternal depression** |
| --- | --- | --- | --- |
| ABCD | Center for Epidemiologic Studies Depression scale (binarized at ≥20) in the first and second trimester | - | Center for Epidemiologic Studies Depression scale (binarized at ≥16) assessed at 4 months after childbirth |
| ALSPAC | Edinburgh Postnatal Depression Scale (binarized at >12) in the second and third trimester | Self-reported depression prior to pregnancy | Edinburgh Postnatal Depression Scale (binarized at >12) assessed at 8 months after childbirth |
| DNBC | Self-reported depression during pregnancy in the first and third trimester | Self-reported depression prior to pregnancy | Items from Symptom Distress Checklist (SCL-90) or the General Health Questionnaire (GHQ 60) assessed at 6 months after childbirth |
| EDEN | Center for Epidemiologic Studies Depression scale (binarized at ≥20) in the second trimester | - | Edinburgh Postnatal Depression Scale (binarized at >12) assessed at 12 months after childbirth |
| Generation R | Brief symptoms inventory (binarized at >0·75) in the second trimester | Self-reported depression prior to pregnancy | Edinburgh Postnatal Depression Scale (binarized at >12) assessed at 2 months after childbirth |
| NINFEA | Self-reported doctor-diagnosed depression at any time during pregnancy | Self-reported doctor-diagnosed depression prior to pregnancy | Self-reported post-partum depression assessed at 18 months after childbirth |
| PREDO | Center for Epidemiologic Studies Depression scale (binarized at ≥20) assessed repeatedly for up to 14 times throughout pregnancy | - | Center for Epidemiologic Studies Depression scale (binarized at ≥20) assessed at 2 weeks and 6 months after childbirth |

# Table S1. Assessment tools for exposure measures per cohort

| **Cohort** | **Internalising symptoms** | **Externalising symptoms** | **ADHD symptoms** | **ASD symptoms** | **Gross motor skills** | **Fine motor skills** | **Non-verbal intelligence** | **Language** |
| --- | --- | --- | --- | --- | --- | --- | --- | --- |
| ABCD | Strengths and Difficulties Questionnaire | Strengths and Difficulties Questionnaire | Strengths and Difficulties Questionnaire | - | - | - | Raven’s Progressive Matrices* | - |
| ALSPAC | Strengths and Difficulties Questionnaire | Strengths and Difficulties Questionnaire | Strengths and Difficulties Questionnaire | - | Denver Developmental Screening Test | Denver Developmental Screening Test | Griffiths Mental Development scales* | Denver Developmental Screening Test |
| DNBC | Strengths and Difficulties Questionnaire** | Strengths and Difficulties Questionnaire** | Strengths and Difficulties Questionnaire** | - | Developmental Coordination Disorder Questionnaire | Developmental Coordination Disorder Questionnaire | - | - |
| EDEN | Strengths and Difficulties Questionnaire | Strengths and Difficulties Questionnaire | Strengths and Difficulties Questionnaire | - | Brunet-Lezine psychometric scale | Brunet-Lezine psychometric scale | Wechsler Preschool and Primary Scale of Intelligence* | Brunet-Lezine psychometric scale |
| GenR | Child Behaviour Checklist | Child Behaviour Checklist | Revised Conners' Parent Rating Scale | Social Responsiveness Scale | Child Development Inventory | Child Development Inventory | Snijders-Oomen Non-Verbal Intelligence Test* | MacArthur Communicative Development Inventory |
| NINFEA | Strengths and Difficulties Questionnaire | Strengths and Difficulties Questionnaire | Diagnostic and Statistical Manual of Mental Disorders, Fourth Edition (4 yrs) and Strengths and Difficulties Questionnaire (13 yrs) | - | Denver Development Screening Test | Denver Development Screening Test | - | Denver Development Screening Test |
| PREDO | Child Behaviour Checklist | Child Behaviour Checklist | The ADHD Rating Scale-IV Preschool Version | Autism Spectrum Screening Questionnaire | - | - | - | - |

# Table S2. Assessment tools for outcome measures per cohort

*Note: ** Assessed by examiner or test performed by child. **Parent-reported, except for the assessment in late adolescence. All other outcomes were reported by parents. All outcomes were investigated on a continuous scale of percentile scores calculated per cohort.

# Table S3. Study characteristics – outcome variables

|  | ABCD  (the Netherlands)  2003-2004 | ALSPAC  (UK)  1991-1992 | DNBC (Denmark)  1995-2002 | EDEN (France)  2003-2006 | Generation R  (the Netherlands)  2002-2006 | NINFEA (Italy)  2005-2016 | PREDO (Finland)  2006-2010 |
| --- | --- | --- | --- | --- | --- | --- | --- |
| **Outcome characteristics** |  |  |  |  |  |  |  |
| *n* (%) with internalising outcome | 4119 (100·0) | 6899 (91·7) | 49559 (99·9) | 1395 (83·7) | 4537 (93·9) | 1319 (22·3) | 1979 (99·3) |
| Age at internalising measurement, years, mean (SD) | 7·6 (3·1) | 9·5 (3·9)* | 11·7 (4·6)* | 5·3 (2·0)* | 5·1 (3·1)* | 13·3 (0·3) | 9·4 (0·8) |
| Internalising percentile score, mean (SD) | 41·6 (32·9) | 42·6 (31·6) | 43·8 (31·4) | 50·5 (28·6) | 53·7 (27·0) | 45·4 (30·2) | 47·2 (30·4) |
| *n* (%) with externalising outcome | 4118 (99·9) | 6898 (91·7) | 49562 (99·9) | 1394 (83·6) | 4544 (94·0) | 1320 (22·3) | 1976 (99·2) |
| Age at externalising measurement, years, mean (SD) | 7·6 (3·1) | 9·5 (3·9)* | 11·7 (4·6)* | 5·3 (2·0)* | 5·1 (3·1)* | 13·3 (0·3) | 9·4 (0·8) |
| Externalising percentile score, mean (SD) | 44·9 (30·8) | 45·0 (29·7) | 44·9 (30·4) | 50·5 (28·8) | 53·1 (27·2) | 45·6 (29·9) | 46·5 (31·1) |
| *n* (%) with ADHD outcome | 4120 (100·0) | 6898 (91·7) | 49561 (99·9) | 1394 (83·6) | 3029 (62·7) | 4728 (79·9) | 1986 (99·7) |
| Age at ADHD measurement, years, mean (SD) | 7·6 (3·1) | 9·5 (3·9)* | 11·7 (4·6)* | 5·3 (2·0)* | 8·2 (0·2) | 6·1 (3·8)* | 9·4 (0·8) |
| ADHD percentile score, mean (SD) | 43·0 (31·5) | 43·3 (29·8) | 43·0 (31·0) | 50·5 (28·6) | 52·8 (27·3) | 44·6 (30·8) | 48·2 (29·4) |
| *n* (%) with ASD outcome | - | - | - | - | 3292 (68·1) | - | 1985 (99·6) |
| Age at ASD measurement, years, mean (SD) | - | - | - | - | 6·2 (0·5) | - | 9·4 (0·8) |
| ASD percentile score, mean (SD) | - | - | - | - | 54·9 (26·8) | - | 43·1 (33·4) |
| *n* (%) with fine motor skills outcome | - | 6093 (81·0) | 38280 (77·2) | 1658 (99·5) | 3941 (81·6) | 5052 (85·4) | - |
| Age at fine motor measurement, years, mean (SD) | - | 1·9 (1·2)* | 7·0 (0·0) | 2·8 (1·7)* | 1·5 (0·5)* | 0·6 (0·1) | - |
| Fine motor percentile score, mean (SD) | - | 47·8 (29·0) | 43·7 (23·1) | 50·6 (27·4) | 64·8 (30·1) | 35·0 (34·2) | - |
| *n* (%) with gross motor skills outcome | - | 5972 (79·4) | 38280 (77·2) | 1660 (99·6) | 3941 (81·6) | 5862 (99·1) | - |
| Age at gross motor measurement, years, mean (SD) | - | 1·9 (1·2)* | 7·0 (0·0) | 2·8 (1·7)* | 1·5 (0·5)* | 2·2 (1·8)* | - |
| Gross motor percentile score, mean (SD) | - | 46·1 (28·3) | 43·5 (23·0) | 50·5 (26·6) | 62·2 (33·4) | 37·9 (30·7) | - |
| *n* (%) with language skills outcome | - | 6562 (87·2) | - | 1661 (99·6) | 3411 (70·6) | 5629 (95·2) | - |
| Age at language skills measurement, years, mean (SD) | - | 3·4 (3·4)* | - | 2·6 (1·8)* | 1·5 (0·1) | 0·6 (0·1) | - |
| Language percentile score, mean (SD) | - | 49·5 (29·4) | - | 50·2 (28·7) | 41·5 (29·9) | 41·8 (29·5) | - |
| *n* (%) with non-verbal intelligence outcome | 1625 (39·4) | 4899 (65·1) | - | 1427 (85·6) | 3599 (74·5) | - | - |
| Age at non-verbal intelligence measurement, years, mean (SD) | 11·1 (0·3) | 8·0 (1·6)* | - | 4·3 (1·1)* | 6·2 (0·5) | - | - |
| Non-verbal intelligence percentile score, mean (SD) | 49·4 (28·8) | 50·8 (28·8) | - | 50·4 (27·3) | 56·2 (29·3) | - | - |
|  |  |  |  |  |  |  |  |

*Note*: Characteristics of the participating study population are based on complete data on the exposure, covariates, and at least one outcome. Calendar years below cohort names indicate the recruitment period. Total sample size = 76,514 children with complete data on exposure (prenatal maternal depression), covariates, and at least one outcome. * Repeated measures (see Figure 1 for details). Mean age and mean percentile scores are reported across all repeated measures if multiple outcome assessments were available.

|  | ABCD  (the Netherlands)  2003-2004 | ALSPAC  (UK)  1991-1992 | DNBC (Denmark)  1995-2002 | EDEN (France)  2003-2006 | Generation R  (the Netherlands)  2002-2006 | NINFEA (Italy)  2005-2016 | PREDO (Finland)  2006-2010 |
| --- | --- | --- | --- | --- | --- | --- | --- |
| n (% of original sample) | 8252 (66·7) | 4498 (37·4%) | 47219 (48·8) | 335 (16·7) | 5069 (51·2) | 1727 (22·6) | 2793 (58·4%) |
| ***Child characteristics*** |  |  |  |  |  |  |  |
| Assigned sex, male, n (%) | 3879 (51·8%, missing: 9·3%) | 2365 (52·6%, missing: 0·0%) | 25023 (53·0%, missing: 0·0%) | 127 (53·8%, missing: 29·6%) | 2536 (51·6%, missing: 3·1%) | 443 (49·4%, missing: 48·1%) | 1481 (53·4%, missing: 0·8%) |
| ***Maternal characteristics*** |  |  |  |  |  |  |  |
| Mother born abroad, yes, n (%) | 2135 (52·1%, missing: 50·4%) | 174 (5·0%, missing: 22·0%) | - | 22 (8·8%, missing: 25·7%) | 1849 (42·5%, missing: 14·1%) | 101 (5·8%, missing: 0·0%) | - |
| High maternal education level, n (%) | 1560 (38·4%, missing: 50·8%) | 312 (7·9%, missing: 12·6%) | 6507 (41·9%, missing: 67·1%) | 90 (37·0%, missing: 27·5%) | 1336 (34·9%, missing: 24·5%) | 835 (50·3%, missing: 3·8%) | 1297 (54·0%, missing: 13·9%) |
| Prenatal maternal depression, n (%) | 891 (22·2%, missing: 51·4%) | 943 (22·9%, missing: 8·3%) | 414 (1·2%, missing: 27·2%) | 53 (21·4%, missing: 26·0%) | 278 (14·8%, missing: 62·9%) | 12 (0·9%, missing: 20·3%) | 340 (24·1%, missing: 49·5%) |
| Postnatal maternal depression, n (%) | 313 (20·3%, missing: 81·3%) | 373 (9·9%, missing: 16·2%) | 4972 (17·3%, missing: 39·1%) | 5 (9·4%, missing: 84·2%) | 167 (10·1%, missing: 67·5%) | 19 (4·2%, missing: 73·8%) | 243 (17·8%, missing: 51·2%) |
| Pre-pregnancy depression, n (%) |  | 402 (10·0%, missing: 10·7%) | 1365 (3·3%, missing: 13·1%) | - | 373 (18·9%, missing: 61·1%) | 47 (3·4%, missing: 20·1%) | - |
| Maternal depression at more than one time points, n (%) |  | 367 (8·7%, missing: 6·3%) | 620 (1·6%, missing: 19·4%) | - | 143 (7·5%, missing: 62·2%) | 11 (0·8%, missing: 20·3%) | - |
| Any alcohol use in pregnancy, yes, n (%) | 768 (18·6%, missing: 49·8%) | 2040 (82·8%, missing: 45·2%) | 23858 (53·8%, missing: 6·1%) | 109 (42·4%, missing: 23·3%) | 1360 (43·6%, missing: 38·4%) | 311 (55·7%, missing: 67·7%) | 242 (15·2%, missing: 43·1%) |
| Any smoking in pregnancy, yes, n (%) | 430 (10·4%, missing: 49·9%) | 1231 (38·0%, missing: 28·0%) | 12957 (29·1%, missing: 5·8%) | 101 (39·9%, missing: 24·5%) | 1018 (29·0%, missing: 30·8%) | 168 (10·3%, missing: 5·8%) | 295 (10·7%, missing: 1·1%) |
| Pre-pregnancy BMI, mean (SD) | 23·3 (4·3) | 23·04 (4·07) | 23·6 (4·4) | 23·8 (5·2) | 24·1 (4·7) | 22·8 (4·1) | 24·86 (5·35) |

# Table S4. Study characteristics of excluded participants per cohort due to missing data on exposure, outcomes and/or confounders.

*Note*: Characteristics of the excluded study population (inclusion was based on complete data on the exposure, covariates, and at least one outcome). Calendar years below cohort names indicate the recruitment period.

# Table S5. Interaction between prenatal maternal depression and child’s assigned sex at birth – internalising, externalising, ADHD and ASD outcomes.

|  | **Internalising symptoms** | | | **Externalising symptoms** | | | **ADHD symptoms** | | | **ASD symptoms** | | |
| --- | --- | --- | --- | --- | --- | --- | --- | --- | --- | --- | --- | --- |
| Cohort | N | β | 95% CI | N | β | 95% CI | N | β | 95% CI | N | β | 95% CI |
| ABCD | 4119 | -3·35 | [-8·04, 1·34] | 4118 | -2·19 | [-6·64, 2·26] | 4120 | -3·90 | [-8·47, 0·66] | - | - | - |
| ALSPAC | 6899 | -0·09 | [-3·06, 2·87] | 6898 | 1·94 | [-1·01, 4·89] | 6898 | 1·88 | [-1·04, 4·8] | - | - | - |
| DNBC | 49562 | -0·73 | [-5·98, 4·52] | 49562 | 2·26 | [-2·94, 7·47] | 49561 | 1·33 | [-3·95, 6·6] | - | - | - |
| EDEN | 1394 | -9·00 | [-16·91, -1·10] | 1394 | -0·24 | [-8·40, 7·92] | 1394 | -2·33 | [-10·25, 5·59] | - | - | - |
| GenR | 4544 | -0·14 | [-4·79, 4·51] | 4544 | -1·07 | [-5·86, 3·73] | 3029 | 1·31 | [-6·44, 9·06] | 3292 | -0·16 | [-7·24, 6·92] |
| NINFEA | 1320 | -1·22 | [-24·10, 21·65] | 1320 | -2·41 | [-25·14, 20·32] | 4728 | 1·74 | [-10·47, 13·94] | - | - | - |
| PREDO | 1979 | -3·67 | [-10·09, 2·76] | 1976 | 0·85 | [-5·71, 7·41] | 1986 | -2·59 | [-8·72, 3·55] | 1985 | -3·89 | [-10·83, 3·05] |
| **TOTAL** | **69807** | **-1·52** | **[-3·39, 0·36]** | **69812** | **0·56** | **[-1·31, 2·43]** | **71716** | **-0·26** | **[-2·64, 2·13]** | **5277** | **-2·06** | **[-7·02, 2·89]** |

*Note*: This model adjusted maternal age at birth, maternal smoking and alcohol consumption during pregnancy, pre-pregnancy maternal BMI, maternal education, the child’s assigned sex at birth and age at outcome assessment. The reference category is assigned male sex at birth. The exposure variable is a binary measure of prenatal depression. The column N refers to the number of observations included per analysis. The I-squared statistic per analysis was: 0·0% for internalising symptoms, 0·0% for externalising symptoms, 22·2% for ADHD symptoms, and 0·0% for ASD symptoms.

# Table S6. Interaction between prenatal maternal depression and child’s assigned sex at birth – motor, language, and non-verbal intelligence outcomes.

|  | **Fine motor skills** | | | **Gross motor skills** | | | **Language skills** | | | **Non-verbal intelligence** | | |
| --- | --- | --- | --- | --- | --- | --- | --- | --- | --- | --- | --- | --- |
| Cohort | N | β | 95% CI | N | β | 95% CI | N | β | 95% CI | N | β | 95% CI |
| ABCD | - | - | - | - | - | - | - | - | - | 1625 | -3·04 | [-11·26, 5·18] |
| ALSPAC | 6093 | -2·06 | [-5·11, 1·00] | 5972 | -1·17 | [-4·10, 1·76] | 6562 | 2·58 | [-0·26, 5·41] | 4899 | 0·68 | [-3·40, 4·75] |
| DNBC | 38280 | -5·44 | [-10·97, 0·08] | 38280 | -4·14 | [-9·61, 1·33] | - | - | - | - | - | - |
| EDEN | 1658 | -1·23 | [-6·38, 3·92] | 1660 | -1·55 | [-6·78, 3·67] | 1661 | -1·77 | [-7·60, 4·06] | 1427 | 0·30 | [-6·38, 6·98] |
| GenR | 3900 | 3·45 | [-2·32, 9·23] | 3908 | -2·12 | [-8·51, 4·26] | 3411 | 4·64 | [-3·49, 12·78] | 3599 | 4·79 | [-1·69, 11·27] |
| NINFEA | 5052 | 3·00 | [-10·60, 16·60] | 5862 | 2·18 | [-7·00, 11·35] | 5629 | 4·78 | [-6·18, 15·73] | - | - | - |
| **TOTAL** | **54983** | **-1·38** | **[-4·03, 1·26]** | **55682** | **-1·61** | **[-3·72, 0·51]** | **17263** | **2·13** | **[-0·24, 4·51]** | **11550** | **0·96** | **[-1·91, 3·83]** |

*Note*: This model adjusted maternal age at birth, maternal smoking and alcohol consumption during pregnancy, pre-pregnancy maternal BMI, maternal education, the child’s assigned sex at birth and age at outcome assessment. The reference category is assigned male sex at birth. The exposure variable is a binary measure of prenatal depression. The column N refers to the number of observations included per analysis. The I-squared statistic per analysis was: 21·7% for fine motor skills, 0·0% for gross motor skills, 0·0% for language skills, and 0·0% for non-verbal intelligence.

# Table S7. Associations between prenatal depression and internalising symptoms via direct and indirect pathways mediated by postnatal depression.

|  | Direct Effect | | | Indirect Effect | | | Perc. Med. |
| --- | --- | --- | --- | --- | --- | --- | --- |
| Cohort | N | β | 95% CI | N | β | 95% CI |  |
| ABCD | 3517 | 6·44 | [3·13, 9·76] | 3517 | 3·52 | [2·29, 4·76] | 35·0% |
| ALSPAC | 5967 | 8·75 | [6·45, 11·05] | 5967 | 1·88 | [1·17, 2·59] | 17·8% |
| DNBC | 32098 | 5·65 | [1·24, 10·05] | 32098 | 3·30 | [2·55, 4·04] | 36·8% |
| EDEN | 1110 | 4·58 | [-0·76, 9·92] | 1110 | 1·57 | [0·33, 2·82] | 24·9% |
| GenR | 2865 | 8·29 | [4·13, 12·46] | 2865 | 2·42 | [1·09, 3·75] | 22·4% |
| PREDO | 1367 | 9·30 | [5·03, 13·57] | 1367 | 3·38 | [1·69, 5·08] | 26·8% |
| **TOTAL** | **46924** | **7·68** | **[6·24, 9·13]** | **46924** | **2·64** | **[1·95, 3·33]** | **25·6%** |

*Note:* Analyses were adjusted for maternal age at birth, maternal smoking and alcohol consumption during pregnancy, pre-pregnancy maternal BMI, maternal education, the child’s assigned sex at birth and age at outcome assessment. The column N refers to the number of observations included per analysis. The I-squared statistic per analysis was 0·0% for the direct model and 59·6% for the indirect model.

# Table S8. Associations between prenatal depression and externalising symptoms via direct and indirect pathways mediated by postnatal depression.

|  | Direct Effect | | | Indirect Effect | | | Perc. Med. |
| --- | --- | --- | --- | --- | --- | --- | --- |
| Cohort | N | β | 95% CI | N | β | 95% CI |  |
| ABCD | 3517 | 5·33 | [2·41, 8·25] | 3517 | 3·37 | [2·30, 4·45] | 38·4% |
| ALSPAC | 5969 | 6·30 | [4·21, 8·39] | 5969 | 1·38 | [0·74, 2·02] | 18·0% |
| DNBC | 32082 | 1·63 | [-2·19, 5·45] | 32082 | 2·59 | [1·98, 3·20] | 59·4% |
| EDEN | 1109 | 3·18 | [-1·98, 8·34] | 1109 | 0·77 | [-0·24, 1·78] | 16·5% |
| GenR | 2870 | 5·77 | [1·79, 9·75] | 2870 | 1·55 | [0·29, 2·80] | 20·8% |
| PREDO | 1363 | 4·51 | [0·33, 8·69] | 1363 | 2·71 | [1·05, 4·37] | 37·0% |
| **TOTAL** | **46910** | **4·98** | **[3·51, 6·45]** | **46910** | **2·02** | **[1·23, 2·81]** | **28·1%** |

*Note:* Analyses were adjusted for maternal age at birth, maternal smoking and alcohol consumption during pregnancy, pre-pregnancy maternal BMI, maternal education, the child’s assigned sex at birth and age at outcome assessment. The column N refers to the number of observations included per analysis. The I-squared statistic per analysis was 13·8% for the direct model and 76·5% for the indirect model.

# Table S9. Associations between prenatal depression and ADHD symptoms via direct and indirect pathways mediated by postnatal depression.

| Cohort | Direct Effect | | | Indirect Effect | | | Perc. Med. |
| --- | --- | --- | --- | --- | --- | --- | --- |
|  | N | β | 95% CI | N | β | 95% CI |  |
| ABCD | 3519 | 4·81 | [1·78, 7·84] | 3519 | 2·51 | [1·44, 3·58] | 33·8% |
| ALSPAC | 6174 | 6·95 | [4·90, 9·00] | 6174 | 1·01 | [0·37, 1·65] | 12·6% |
| DNBC | 32081 | 1·44 | [-2·32, 5·20] | 32081 | 2·12 | [1·58, 2·66] | 56·5% |
| EDEN | 1293 | 0·46 | [-4·19, 5·11] | 1293 | 0·85 | [-0·10, 1·80] | 22·9% |
| GenR | 2246 | 9·25 | [3·88, 14·61] | 2246 | 1·01 | [-0·77, 2·80] | 9·6% |
| PREDO | 1373 | 3·82 | [-0·12, 7·77] | 1373 | 3·99 | [2·35, 5·62] | 50·5% |
| **TOTAL** | **46686** | **4·52** | **[2·16, 6·88]** | **46686** | **1·84** | **[0·98, 2·69]** | **28·2%** |

*Note:* Analyses were adjusted for maternal age at birth, maternal smoking and alcohol consumption during pregnancy, pre-pregnancy maternal BMI, maternal education, the child’s assigned sex at birth and age at outcome assessment. The column N refers to the number of observations included per analysis. The I-squared statistic per analysis was 62·1% for the direct model and 79·8% for the indirect model.

# Table S10. Associations between prenatal depression and ASD symptoms via direct and indirect pathways mediated by postnatal depression.

| Cohort | Direct Effect | | | Indirect Effect | | | Perc. Med. |
| --- | --- | --- | --- | --- | --- | --- | --- |
|  | N | β | 95% CI | N | β | 95% CI |  |
| GenR | 2432 | 5·53 | [0·88, 10·18] | 2432 | 2·33 | [0·92, 3·74] | 28·9% |
| PREDO | 1372 | 5·45 | [1·30, 9·61] | 1372 | 3·24 | [1·52, 4·96] | 37·2% |
| **TOTAL** | **3804** | **5·49** | **[2·39, 8·59]** | **3804** | **2·70** | **[1·60, 3·79]** | **32·4%** |

*Note:* Analyses were adjusted for maternal age at birth, maternal smoking and alcohol consumption during pregnancy, pre-pregnancy maternal BMI, maternal education, the child’s assigned sex at birth and age at outcome assessment. The column N refers to the number of observations included per analysis. The I-squared statistic per analysis was 0·0% for the direct and indirect model.

# Table S11. Association between prenatal maternal depression and internalising, externalising, ADHD and ASD outcomes, adjusted for assigned sex and age at outcome assessment.

|  | **Internalising symptoms** | | | **Externalising symptoms** | | | **ADHD symptoms** | | | **ASD symptoms** | | |
| --- | --- | --- | --- | --- | --- | --- | --- | --- | --- | --- | --- | --- |
| Cohort | N | β | 95% CI | N | β | 95% CI | N | β | 95% CI | N | β | 95% CI |
| ABCD | 4119 | 11·49 | [9·09, 13·88] | 4118 | 10·47 | [8·20, 12·74] | 4120 | 9·53 | [7·20, 11·85] | - | - | - |
| ALSPAC | 6899 | 12·18 | [10·98, 13·38] | 6898 | 10·24 | [9·04, 11·44] | 6898 | 9·11 | [7·93, 10·29] | - | - | - |
| DNBC | 49559 | 12·35 | [9·68, 15·02] | 49562 | 6·80 | [4·13, 9·46] | 49561 | 6·10 | [3·41, 8·79] | - | - | - |
| EDEN | 1395 | 7·88 | [3·90, 11·87] | 1394 | 5·76 | [1·58, 9·95] | 1394 | 3·23 | [-0·87, 7·33] | - | - | - |
| GenR | 4537 | 14·02 | [11·69, 16·36] | 4544 | 12·31 | [9·90, 14·72] | 3029 | 10·05 | [6·16, 13·93] | 3292 | 11·29 | [7·65, 14·92] |
| NINFEA | 1319 | 16·32 | [4·93, 27·72] | 1320 | 15·29 | [3·95, 26·63] | 4728 | 10·50 | [4·32, 16·68] | - | - | - |
| PREDO | 1995 | 14·47 | [11·26, 17·69] | 1992 | 9·08 | [5·79, 12·36] | 2002 | 9·68 | [6·61, 12·76] | 2001 | 11·00 | [7·48, 14·53] |
| **TOTAL** | **69823** | **12·36** | **[11·36, 13·36]** | **69828** | **9·58** | **[7·85, 11·30]** | **71732** | **8·37** | **[6·76, 9·98]** | **5293** | **11·14** | **[8·61, 13·67]** |

*Note*: The exposure variable is a binary measure of prenatal depression. The column N refers to the number of observations included per analysis. The I-squared statistic per analysis was: 13·1% for internalising symptoms, 63·9% for externalising symptoms, 55·5% for ADHD symptoms, and 0·0% for ASD symptoms.

# Table S12. Associations between prenatal maternal depression and motor, language, and non-verbal intelligence outcomes, adjusted for assigned sex and age at outcome assessment.

|  | **Fine motor skills** | | | **Gross motor skills** | | | **Language skills** | | | **Non-verbal intelligence** | | |
| --- | --- | --- | --- | --- | --- | --- | --- | --- | --- | --- | --- | --- |
| Cohort | N | β | 95% CI | N | β | 95% CI | N | β | 95% CI | N | β | 95% CI |
| ABCD | - | - | - | - | - | - | - | - | - | 1625 | -1·16 | [-5·32, 3·01] |
| ALSPAC | 6093 | -0·49 | [-1·72, 0·73] | 5972 | 2·16 | [0·99, 3·33] | 6562 | -1·96 | [-3·12, -0·80] | 4899 | -5·35 | [-7·08, -3·62] |
| DNBC | 38280 | -3·55 | [-6·31, -0·78] | 38280 | -3·50 | [-6·24, -0·77] | - | - | - | - | - | - |
| EDEN | 1658 | 0·57 | [-2·03, 3·16] | 1660 | 1·48 | [-1·14, 4·09] | 1661 | -0·22 | [-3·17, 2·74] | 1427 | -1·22 | [-4·67, 2·24] |
| GenR | 3941 | -0·43 | [-3·31, 2·45] | 3941 | 5·53 | [2·30, 8·76] | 3411 | -1·68 | [-5·76, 2·40] | 3599 | -7·76 | [-11·1, -4·42] |
| NINFEA | 5052 | 5·57 | [-1·25, 12·40] | 5862 | 1·40 | [-3·21, 6·00] | 5629 | 2·15 | [-3·35, 7·65] | - | - | - |
| **TOTAL** | **55024** | **-0·57** | **[-2·10, 0·97]** | **55715** | **1·38** | **[-1·46, 4·22]** | **17263** | **-1·41** | **[-2·71, -0·11]** | **11550** | **-4·07** | **[-7·12, -1·03]** |

*Note*: The exposure variable is a binary measure of prenatal depression. The column N refers to the number of observations included per analysis. The I-squared statistic per analysis was: 42·7% for fine motor skills, 83·7% for gross motor skills, 11·7% for language skills, and 75·1% for non-verbal intelligence.

# Table S13. Associations between prenatal maternal depression and internalising, externalising, ADHD and ASD outcomes, additionally adjusted for maternal country of birth.

|  | **Internalising symptoms** | | | **Externalising symptoms** | | | **ADHD symptoms** | | | **ASD symptoms** | | |
| --- | --- | --- | --- | --- | --- | --- | --- | --- | --- | --- | --- | --- |
| Cohort | N | β | 95% CI | N | β | 95% CI | N | β | 95% CI | N | β | 95% CI |
| ABCD | 4106 | 6·75 | [4·33, 9·17] | 4105 | 7·01 | [4·71, 9·31] | 4107 | 6·54 | [4·17, 8·90] | - | - | - |
| ALSPAC | 6501 | 10·98 | [9·42, 12·54] | 6500 | 8·16 | [6·61, 9·71] | 6500 | 7·36 | [5·83, 8·89] | - | - | - |
| EDEN | 1387 | 7·57 | [3·58, 11·56] | 1386 | 4·23 | [0·12, 8·35] | 1386 | 1·57 | [-2·43, 5·56] | - | - | - |
| GenR | 4536 | 10·98 | [8·61, 13·35] | 4543 | 9·81 | [7·36, 12·26] | 3029 | 9·90 | [5·95, 13·85] | 3292 | 6·83 | [3·25, 10·41] |
| NINFEA | 1319 | 15·51 | [4·09, 26·94] | 1320 | 14·51 | [3·16, 25·86] | 4728 | 9·35 | [3·25, 15·46] | - | - | - |
| **TOTAL** | **17849** | **9·52** | **[7·33, 11·72]** | **17854** | **7·92** | **[6·36, 9·49]** | **19750** | **6·80** | **[4·31, 9·30]** | **3292** | **6·83** | **[3·25, 10·41]** |

*Note*: This model adjusted maternal age at birth, maternal smoking and alcohol consumption during pregnancy, pre-pregnancy maternal BMI, maternal education, maternal country of birth, the child’s assigned sex at birth and age at outcome assessment. The exposure variable is a binary measure of prenatal depression. The column N refers to the number of observations included per analysis. The I-squared statistic per analysis was: 65·0% for internalising symptoms, 36·9% for externalising symptoms, and 69·2% for ADHD symptoms.

# Table S14. Associations between prenatal maternal depression and motor, language, and non-verbal intelligence outcomes, additionally adjusted for maternal country of birth.

|  | **Fine motor skills** | | | **Gross motor skills** | | | **Language skills** | | | **Non-verbal intelligence** | | |
| --- | --- | --- | --- | --- | --- | --- | --- | --- | --- | --- | --- | --- |
| Cohort | N | β | 95% CI | N | β | 95% CI | N | β | 95% CI | N | β | 95% CI |
| ABCD | - | - | - | - | - | - | - | - | - | 1621 | 0·31 | [-3·88, 4·50] |
| ALSPAC | 5735 | -0·90 | [-2·50, 0·69] | 5621 | 1·53 | [0·00, 3·06] | 6181 | -1·73 | [-3·21, -0·25] | 4616 | -3·78 | [-5·92, -1·64] |
| EDEN | 1648 | 1·39 | [-1·22, 4·00] | 1650 | 1·30 | [-1·34, 3·95] | 1651 | 0·86 | [-2·09, 3·81] | 1419 | 0·61 | [-2·77, 3·99] |
| GenR | 3898 | -1·82 | [-4·77, 1·13] | 3906 | 0·62 | [-2·64, 3·87] | 3410 | -3·04 | [-7·20, 1·11] | 3598 | -2·40 | [-5·71, 0·92] |
| NINFEA | 5052 | 5·16 | [-1·65, 11·97] | 5862 | 1·64 | [-2·96, 6·23] | 5629 | 2·86 | [-2·63, 8·34] | - | - | - |
| **TOTAL** | **16333** | **-0·13** | **[-1·88, 1·61]** | **17039** | **1·37** | **[0·18, 2·56]** | **16871** | **-0·79** | **[-2·74, 1·16]** | **11537** | **-1·67** | **[-3·92, 0·58]** |

*Note*: This model adjusted maternal age at birth, maternal smoking and alcohol consumption during pregnancy, pre-pregnancy maternal BMI, maternal education, maternal country of birth, the child’s assigned sex at birth and age at outcome assessment. The exposure variable is a binary measure of prenatal depression. The column N refers to the number of observations included per analysis. The I-squared statistic per analysis was: 36·8% for fine motor skills, 0·0% for gross motor skills, 38·6% for language skills, and 51·1% for non-verbal intelligence.

# Table S15. Associations between continuous prenatal maternal depressive symptoms and internalising, externalising, ADHD and ASD outcomes.

|  | **Internalising symptoms** | | | **Externalising symptoms** | | | **ADHD symptoms** | | | **ASD symptoms** | | |
| --- | --- | --- | --- | --- | --- | --- | --- | --- | --- | --- | --- | --- |
| Cohort | N | β | 95% CI | N | β | 95% CI | N | β | 95% CI | N | β | 95% CI |
| ABCD | 4119 | 5·90 | [4·95, 6·86] | 4118 | 5·15 | [4·24, 6·05] | 4120 | 4·41 | [3·48, 5·34] | - | - | - |
| ALSPAC | 6899 | 6·66 | [6·06, 7·26] | 6898 | 5·06 | [4·45, 5·67] | 6898 | 4·41 | [3·81, 5·01] | - | - | - |
| EDEN | 1395 | 5·26 | [3·89, 6·64] | 1394 | 4·06 | [2·63, 5·48] | 1394 | 2·78 | [1·39, 4·17] | - | - | - |
| GenR | 4537 | 4·92 | [4·17, 5·67] | 4544 | 4·10 | [3·32, 4·88] | 3029 | 3·53 | [2·29, 4·78] | 3292 | 3·00 | [1·88, 4·13] |
| PREDO | 1979 | 8·58 | [7·27, 9·89] | 1976 | 6·55 | [5·20, 7·90] | 1986 | 7·08 | [5·82, 8·34] | 1985 | 6·62 | [5·18, 8·05] |
| **TOTAL** | **18929** | **6·24** | **[5·02, 7·45]** | **18930** | **4·94** | **[4·18, 5·71]** | **17427** | **4·45** | **[3·09, 5·81]** | **5277** | **4·78** | **[1·24, 8·32]** |

*Note*: This model adjusted maternal age at birth, maternal smoking and alcohol consumption during pregnancy, pre-pregnancy maternal BMI, maternal education, the child’s assigned sex at birth and age at outcome assessment. The column N refers to the number of observations included per analysis. The I-squared statistic per analysis was: 88·5% for internalising symptoms, 69·8% for externalising symptoms, 88·8% for ADHD symptoms, and 93·4% for ASD symptoms.

# Table S16. Associations between continuous prenatal maternal depressive symptoms and motor, language, and non-verbal intelligence outcomes.

|  | **Fine motor skills** | | | **Gross motor skills** | | | **Language skills** | | | **Non-verbal intelligence** | | |
| --- | --- | --- | --- | --- | --- | --- | --- | --- | --- | --- | --- | --- |
| Cohort | N | β | 95% CI | N | β | 95% CI | N | β | 95% CI | N | β | 95% CI |
| ABCD | - | - | - | - | - | - | - | - | - | 1625 | -0·10 | [-1·76, 1·55] |
| ALSPAC | 6093 | -0·46 | [-1·09, 0·17] | 5972 | 0·99 | [0·38, 1·61] | 6562 | -1·09 | [-1·68, -0·50] | 4899 | -2·19 | [-3·03, -1·35] |
| EDEN | 1658 | -0·35 | [-1·26, 0·56] | 1660 | 0·32 | [-0·60, 1·25] | 1661 | -0·05 | [-1·08, 0·98] | 1427 | -0·57 | [-1·74, 0·60] |
| GenR | 3900 | -0·64 | [-1·56, 0·29] | 3908 | 1·08 | [0·05, 2·10] | 3411 | -1·10 | [-2·41, 0·20] | 3599 | -1·47 | [-2·51, -0·44] |
| **TOTAL** | **11651** | **-0·47** | **[-0·93, -0·02]** | **11540** | **0·84** | **[0·38, 1·30]** | **11634** | **-0·80** | **[-1·46, -0·14]** | **11550** | **-1·23** | **[-2·14, -0·33]** |

*Note*: This model adjusted maternal age at birth, maternal smoking and alcohol consumption during pregnancy, pre-pregnancy maternal BMI, maternal education, the child’s assigned sex at birth and age at outcome assessment. The column N refers to the number of observations included per analysis. The I-squared statistic per analysis was: 0·0% for fine motor skills, 0·0% for gross motor skills, 35·8% for language skills, and 60·9% for non-verbal intelligence.

# Table S17. Individual associations between pre-pregnancy depression and internalising, externalising, ADHD and ASD outcomes.

|  | **Internalising symptoms** | | | **Externalising symptoms** | | | **ADHD symptoms** | | | **ASD symptoms** | | |
| --- | --- | --- | --- | --- | --- | --- | --- | --- | --- | --- | --- | --- |
| Cohort | N | β | 95% CI | N | β | 95% CI | N | β | 95% CI | N | β | 95% CI |
| ALSPAC | 6899 | 9·07 | [6·89, 11·24] | 6898 | 6·68 | [4·52, 8·83] | 6898 | 6·17 | [4·05, 8·30] | - | - | - |
| DNBC | 52308 | 8·54 | [7·19, 9·88] | 52310 | 4·17 | [2·83, 5·51] | 52309 | 4·26 | [2·90, 5·62] | - | - | - |
| GenR | 4772 | 7·32 | [5·80, 8·84] | 4776 | 6·54 | [4·96, 8·12] | 3188 | 7·75 | [5·37, 10·13] | 3482 | 2·25 | [0·08, 4·41] |
| NINFEA | 1349 | 7·41 | [-1·61, 16·43] | 1350 | 10·74 | [1·80, 19·68] | 4992 | 9·00 | [4·46, 13·54] | - | - | - |
| **TOTAL** | **65328** | **8·18** | **[7·24, 9·13]** | **65334** | **5·85** | **[4·17, 7·54]** | **67387** | **6·29** | **[4·31, 8·27]** | **3482** | **2·25** | **[0·08, 4·41]** |

*Note*: This model adjusted maternal age at birth, pre-pregnancy maternal BMI, maternal education, the child’s assigned sex at birth and age at outcome assessment. The exposure variable is a binary measure of pre-pregnancy depression. The column N refers to the number of observations included per analysis. The I-squared statistic per analysis was: 4·3% for internalising symptoms, 59·8% for externalising symptoms, and 66·2% for ADHD symptoms.

# Table S18. Individual associations between postnatal depression and internalising, externalising, ADHD and ASD outcomes.

|  | **Internalising symptoms** | | | **Externalising symptoms** | | | **ADHD symptoms** | | | **ASD symptoms** | | |
| --- | --- | --- | --- | --- | --- | --- | --- | --- | --- | --- | --- | --- |
| Cohort | N | β | 95% CI | N | β | 95% CI | N | β | 95% CI | N | β | 95% CI |
| ABCD | 3664 | 12·10 | [9·47, 14·73] | 3664 | 11·74 | [9·22, 14·25] | 3665 | 9·55 | [6·96, 12·15] | - | - | - |
| ALSPAC | 6899 | 13·42 | [11·23, 15·62] | 6898 | 9·28 | [7·09, 11·48] | 6898 | 8·09 | [5·93, 10·26] | - | - | - |
| DNBC | 43299 | 8·26 | [7·61, 8·92] | 43301 | 6·30 | [5·65, 6·95] | 43300 | 5·66 | [4·99, 6·32] | - | - | - |
| EDEN | 1302 | 13·52 | [8·62, 18·42] | 1301 | 9·74 | [4·69, 14·79] | 1301 | 5·52 | [0·62, 10·42] | - | - | - |
| GenR | 3582 | 10·36 | [7·62, 13·10] | 3588 | 7·44 | [4·59, 10·29] | 2513 | 6·82 | [2·38, 11·26] | 2706 | 10·99 | [6·89, 15·09] |
| NINFEA | - | - | - | - | - | - | 3200 | 5·64 | [-0·97, 12·26] | - | - | - |
| PREDO | 1956 | 15·04 | [11·44, 18·64] | 1953 | 12·04 | [8·39, 15·69] | 1963 | 14·12 | [10·72, 17·53] | 1962 | 13·06 | [9·20, 16·92] |
| **TOTAL** | **61004** | **11·59** | **[9·43, 13·75]** | **61007** | **8·99** | **[6·96, 11·02]** | **62840** | **8·08** | **[5·74, 10·43]** | **4668** | **12·09** | **[9·28, 14·90]** |

*Note*: This model adjusted maternal age at birth, maternal smoking and alcohol consumption during pregnancy, pre-pregnancy maternal BMI, maternal education, the child’s assigned sex at birth and age at outcome assessment. The exposure variable is a binary measure of postnatal depression. The column N refers to the number of observations included per analysis. The I-squared statistic per analysis was: 78·4% for internalising symptoms, 75·2% for externalising symptoms, 81·3% for ADHD symptoms, and 0·0% for ASD symptoms.

# Table S19. Leave-one-out analyses for the associations between prenatal depression and internalising, externalising, ADHD, and ASD symptoms.

|  | **Internalising symptoms** | | | **Externalising symptoms** | | | **ADHD symptoms** | | | **ASD symptoms** | | |
| --- | --- | --- | --- | --- | --- | --- | --- | --- | --- | --- | --- | --- |
| Cohort | N | β | 95% CI | N | β | 95% CI | N | β | 95% CI | N | β | 95% CI |
| Excl. ABCD | 65688 | 11·41 | [10·37, 12·44] | 65694 | 7·59 | [5·57, 9·61] | 67596 | 6·67 | [4·31, 9·03] | - | - | - |
| Excl ALSPAC | 62908 | 10·84 | [8·74, 12·94] | 62914 | 7·42 | [5·34, 9·49] | 64818 | 6·46 | [4·07, 8·84] | - | - | - |
| Excl. DNBC | 20248 | 10·84 | [8·86, 12·82] | 20250 | 8·11 | [6·85, 9·37] | 22155 | 7·04 | [5·06, 9·03] | - | - | - |
| Excl. EDEN | 68412 | 11·25 | [9·69, 12·81] | 68418 | 7·92 | [6·36, 9·47] | 70322 | 7·12 | [5·74, 8·5] | - | - | - |
| Excl. GenR | 65270 | 10·68 | [8·76, 12·61] | 65268 | 7·07 | [5·69, 8·46] | 68687 | 6·25 | [4·29, 8·21] | 1985 | 9·58 | [6·09, 13·06] |
| Excl. NINFEA | 68488 | 10·81 | [9·21, 12·41] | 68492 | 7·43 | [5·83, 9·03] | 66988 | 6·41 | [4·43, 8·38] | - | - | - |
| Excl. PREDO | 67828 | 10·47 | [9·02, 11·91] | 67836 | 7·44 | [5·55, 9·33] | 69730 | 6·21 | [4·23, 8·19] | 3292 | 8·07 | [4·48, 11·65] |

*Note:* Analyses were adjusted for maternal age at birth, maternal smoking and alcohol consumption during pregnancy, pre-pregnancy maternal BMI, maternal education, the child’s assigned sex at birth and age at outcome assessment. The column N refers to the number of observations included per analysis.

# Table S20. Leave-one-out analyses for the associations between prenatal depression and motor, language, and non-verbal intelligence outcomes.

|  | **Fine motor skills** | | | **Gross motor skills** | | | **Language skills** | | | **Non-verbal intelligence** | | |
| --- | --- | --- | --- | --- | --- | --- | --- | --- | --- | --- | --- | --- |
| Cohort | N | β | 95% CI | N | β | 95% CI | N | β | 95% CI | N | β | 95% CI |
| Excl. ABCD | - | - | - | - | - | - | - | - | - | 9925 | -2·55 | [-5·28, 0·18] |
| Excl. ALSPAC | 48890 | -0·35 | [-3·52, 2·81] | 49710 | 0·16 | [-2·64, 2·96] | 10701 | -0·01 | [-3·12, 3·10] | 6651 | -0·95 | [-3·83, 1·92] |
| Excl. DNBC | 16703 | -0·07 | [-1·68, 1·53] | 17402 | 1·67 | [0·51, 2·84] | - | - | - | - | - | - |
| Excl. EDEN | 53325 | -1·34 | [-3·30, 0·61] | 54022 | 0·31 | [-2·46, 3·09] | 15602 | -1·76 | [-3·08, -0·44] | 10123 | -2·82 | [-5·25, -0·39] |
| Excl. GenR | 51083 | -0·31 | [-3·25, 2·63] | 51774 | 0·26 | [-2·40, 2·92] | 13852 | -0·20 | [-2·83, 2·42] | 7951 | -1·28 | [-4·53, 1·97] |
| Excl. NINFEA | 49931 | -1·10 | [-3·05, 0·85] | 49820 | 0·38 | [-2·18, 2·94] | 11634 | -1·37 | [-3·26, 0·52] | - | - | - |

*Note:* Analyses were adjusted for maternal age at birth, maternal smoking and alcohol consumption during pregnancy, pre-pregnancy maternal BMI, maternal education, the child’s assigned sex at birth and age at outcome assessment. The column N refers to the number of observations included per analysis.

# Table S21. Associations between prenatal maternal depression ADHD outcomes based on maternal and teacher report (Generation R only).

|  | **ADHD symptoms** | | |
| --- | --- | --- | --- |
| Rater | N | β | 95% CI |
| Mother report | 3029 | 9·31 | [5·37, 13·26] |
| Teacher report | 2648 | 4·50 | [2·98, 6·02] |

*Note*: Analyses adjusted for maternal age at birth, maternal smoking and alcohol consumption during pregnancy, pre-pregnancy maternal BMI, maternal education, maternal country of birth, the child’s assigned sex at birth and age at outcome assessment. The exposure variable is a binary measure of prenatal depression. The maternal report is from the Revised Conners' Parent Rating Scale at child age 8 years and the teacher report is from the Attention scale of the Child Behaviour Checklist at child age 6 years.

# References

1. van Eijsden M, Vrijkotte TGM, Gemke RJBJ, van der Wal MF. Cohort profile: the Amsterdam Born Children and their Development (ABCD) study. Int J Epidemiol. 2011 Oct;40(5):1176–86.

2. Strandberg-Larsen K, Cederkvist L, Aakjær A, Bjerregaard AA, Brix N, Feenstra B, et al. Cohort Profile: the Danish National Birth Cohort from foetal life to young adulthood. Int J Epidemiol. 2025 Jun 11;54(4):dyaf083.

3. Heude B, Forhan A, Slama R, Douhaud L, Bedel S, Saurel-Cubizolles MJ, et al. Cohort Profile: The EDEN mother-child cohort on the prenatal and early postnatal determinants of child health and development. Int J Epidemiol. 2016 Apr;45(2):353–63.

4. Kooijman MN, Kruithof CJ, Van Duijn CM, Duijts L, Franco OH, Van IJzendoorn MH, et al. The Generation R Study: design and cohort update 2017. Eur J Epidemiol. 2016 Dec;31(12):1243–64.

5. Richiardi L, Baussano I, Vizzini L, Douwes J, Pearce N, Merletti F, et al. Feasibility of recruiting a birth cohort through the Internet: the experience of the NINFEA cohort. Eur J Epidemiol. 2007;22(12):831–7.

6. Girchenko P, Lahti M, Tuovinen S, Savolainen K, Lahti J, Binder EB, et al. Cohort Profile: Prediction and prevention of preeclampsia and intrauterine growth restriction (PREDO) study. Int J Epidemiol. 2017 Oct 1;46(5):1380–1381g.

7. Boyd A, Golding J, Macleod J, Lawlor DA, Fraser A, Henderson J, et al. Cohort Profile: the ’children of the 90s’--the index offspring of the Avon Longitudinal Study of Parents and Children. Int J Epidemiol. 2013 Feb;42(1):111–27.

8. Fraser A, Macdonald-Wallis C, Tilling K, Boyd A, Golding J, Davey Smith G, et al. Cohort Profile: the Avon Longitudinal Study of Parents and Children: ALSPAC mothers cohort. Int J Epidemiol. 2013 Feb;42(1):97–110.
